# Individual versus group decision making: Jurors’ reliance on central and peripheral information to evaluate expert testimony

**DOI:** 10.1371/journal.pone.0183580

**Published:** 2017-09-20

**Authors:** Jessica M. Salerno, Bette L. Bottoms, Liana C. Peter-Hagene

**Affiliations:** 1 School of Social and Behavioral Sciences, Arizona State University, Glendale, AZ, United States of America; 2 Department of Psychology, University of Illinois at Chicago, Chicago, IL, United States of America; 3 Department of Psychology, Southern Illinois University Carbondale, Carbondale, IL, United States of America; Technion Israel Institute of Technology, ISRAEL

## Abstract

To investigate dual-process persuasion theories in the context of group decision making, we studied low and high need-for-cognition (NFC) participants within a mock trial study. Participants considered plaintiff and defense expert scientific testimony that varied in argument strength. All participants heard a cross-examination of the experts focusing on peripheral information (e.g., credentials) about the expert, but half were randomly assigned to also hear central information highlighting flaws in the expert’s message (e.g., quality of the research presented by the expert). Participants rendered pre- and post-group-deliberation verdicts, which were considered “scientifically accurate” if the verdicts reflected the strong (versus weak) expert message, and “scientifically inaccurate” if they reflected the weak (versus strong) expert message. For individual participants, we replicated studies testing classic persuasion theories: Factors promoting reliance on central information (i.e., central cross-examination, high NFC) improved verdict accuracy because they sensitized individual participants to the quality discrepancy between the experts’ messages. Interestingly, however, at the group level, the more that scientifically accurate mock jurors discussed peripheral (versus central) information about the experts, the more likely their group was to reach the scientifically accurate verdict. When participants were arguing for the scientifically accurate verdict consistent with the strong expert message, peripheral comments increased their persuasiveness, which made the group more likely to reach the more scientifically accurate verdict.

## Introduction

When we make decisions, from voting in the upcoming election to buying a new couch, we must evaluate the strength of conflicting persuasive messages and ultimately side with one. Influential dual-process models of persuasive communication (Elaboration Likelihood Model [ELM], [1]; Heuristic-Systematic Model [HSM], [2–3]) assert that focusing on the substantive *content* of a message (i.e., central processing focused on the strength of the message itself) helps individuals evaluate argument strength more accurately than heuristics about the message *source* (i.e., peripheral processing focused on irrelevant or tangential information about the source of the message). Although much work has examined how individuals evaluate conflicting persuasive appeals, tests of such dual-process persuasion models in a group context are rare. Given that American society leaves important decisions to groups, from juries to Congress, it is crucial to examine the process by which *groups* evaluate the strength of conflicting persuasive appeals.

In the context of a legal trial, jurors often must evaluate two opposing expert witnesses delivering conflicting testimony that varies in message strength. We used this context to investigate how mock jurors’ reliance on central and peripheral information about the experts influences their ability to reach accurate decisions about which of two opposing experts to trust. We examined this at both the individual (juror) and group (jury) level. Specifically, at the individual level, we hypothesized that encouraging mock jurors to focus on the central information delivered by expert witnesses would help them evaluate the strength of the witnesses’ messages. Yet at the group level, we had reason to make the novel prediction that complementing central information about the quality of experts’ scientific evidence (i.e., message) during group deliberation with peripheral information about the experts’ credentials (i.e., source) would make jurors favoring the stronger expert more persuasive to their co-jurors, thereby helping the jury reach a consensus decision in line with the more scientifically valid argument; a hypothesis we justify theoretically below.

### Dual process models and individuals

The current investigation is informed by two seminal dual-process models theorizing that people can process persuasive messages through two different routes. The first route is a deliberative, effortful route (i.e., “centrally” according to the ELM, [1], or “systematically” according to the HSM, [2]). The second route is quicker and more efficient (i.e., “peripherally” according to the ELM, or “heuristically” according to the HSM). This tenet is shared conceptually with other dual-process models (System-1/System-2 reasoning, [4–5]). The two routes to persuasion are characterized by one’s reliance on two different types of information. The central (or systematic) route is characterized by reliance on scrutinizing the substantive merit of the actual *message* (e.g., the scientific merit of an expert witness’ testimony) whereas the peripheral (or heuristic) route is characterized by heuristic reliance on contextual or peripheral cues about the *source* of the message (e.g., an expert witness’ credentials) [1].

The ELM posits that people engage in one *or* the other, while the HSM allows for *simultaneous* systematic and heuristic processing—but both models account for the two routes to persuasion and specify factors that encourage reliance on one route versus the other. Factors that increase motivation and/or ability to focus on central, rather than peripheral, information help people assess argument strength more accurately. One such factor is an individual difference factor called need for cognition (NFC, [6]). High NFC people, who are generally inclined to think deeply, are more likely to rely on central information than peripheral information [7–8] and in turn, discriminate better between strong and weak messages (e.g., [9–10]). In contrast, low NFC people, who are less inclined to process a message deeply, are more affected by heuristic (i.e., peripheral) information (e.g., source expertise level, [11]; how much an expert is paid to testify,[12]), which can interfere with accurate assessments of a message’s substantive strength [13].

In court trials, both central and peripheral information about expert testimony can be relevant to jurors’ decisions. For example, when judging expert witnesses’ credibility, jurors are actually instructed to consider some peripheral information, such as the quality of the experts’ credentials [14]. Yet, although peripheral information might be *relevant* to jurors’ assessment of the expert’s overall credibility, prior research suggests that peripheral information can interfere with one’s ability to evaluate whether an expert is presenting a strong or weak message (e.g., when an expert witness bases his or her testimony on a scientifically valid versus invalid study). Specifically, studies have shown that peripheral information can, in fact, distract mock jurors from assessing the quality of the expert’s central message—particularly mock jurors who are prone to rely on peripheral information. For example, manipulations of the validity of scientific procedures presented by an expert witness (i.e., central information) affect judgments made by mock jurors with high, but not low, need for cognition [15]. Conversely, manipulations of source credibility (i.e., peripheral information) affect judgments made by participants with low, but not high, NFC [16]. Contextual factors also influence jurors’ routes to persuasion. For example, when mock jurors hear expert testimony delivered in complex, hard-to-process language versus simple language, they are less likely to recognize flaws in scientific evidence (i.e., central information) and more likely to rely on peripheral information about the experts’ credentials, such as their payment for and frequency of testimony [12, 17].

Thus, we predicted that factors that promote reliance on central information about expert testimony would help individual mock jurors correctly assess the quality of expert testimony. More specifically, we predicted that being (a) intrinsically motivated to rely on central information (i.e., being higher in NFC) and (b) extrinsically prompted to rely on central information about the experts, would both lead to scientifically accurate verdicts in line with the strong expert. We manipulated extrinsic motivation to rely on central information by randomly assigning half of our mock jurors to hear a cross-examination of the weak expert that highlighted flaws in the expert’s message (i.e., the validity of his research).

### Dual-process models and groups

Despite ample research testing dual-process models of persuasion at the individual level, current knowledge falls short when considering group decision making. How do groups use central and peripheral information about dueling experts during deliberation, and how do these arguments shape the quality of a final group decision? The only study we know of that includes prolonged, live group discussion and a unanimity requirement for the final group decision (as juries usually do) found that mock jurors with higher NFC (i.e., who are more inclined to focus on central information) talked more and were perceived as more persuasive than their lower NFC counterparts [18]. This study did not, however, investigate mock jurors’ evaluation of message strength or opinion change (i.e., persuasion).

In other group-decision-making studies, McKimmie and colleagues specifically manipulated central and peripheral information about a persuasion source (in the context of jury decisions, [19]; and in the context of organizational decision making, [20–21]). These authors conclude that their work demonstrates the potential for group deliberation to promote reliance on both central and peripheral information—for example, mock jurors were affected by the gender of an expert only after they deliberated as a group [19]. We note, however, that Rijnbout & McKimmie [20–21] operationalized their central factor as the strength of a job applicant’s Curriculum Vitae (CV). It is difficult to apply this specific central factor manipulation to the context of expert witnesses because—although the strength of an applicant’s CV might be central to the argument being made in that context (i.e., that an applicant is the best candidate for a job)—the strength of an expert’s CV would be peripheral to their in-court message (i.e., testimony that is relevant to the case). In other words, in the context of expert testimony, CV strength would be communicating information about an expert’s credentials, which would be peripheral in this context because credentials (like witness gender) have no bearing specifically on the strength of the central, substantive content of the expert’s testimony. This is true even though credentials might be relevant in considering the expert’s overall credibility. Further, although we learn a lot about group decision making from these studies, the generalizability of these results to trial decision making is somewhat limited because two of the studies included computer simulation of a scripted interaction with confederates rather than actual groups interacting, and the other allowed only 5 minutes of discussion and did not require a unanimous group-level decision.

#### Will group deliberation help or hinder evaluation of message strength?

Group discussion can shift individuals’ opinions to the degree that it provides them with persuasive arguments [22–23], which could help or hinder group evaluation of message strength. On the one hand, deliberation might provide an opportunity for individuals who rely on the central information conveyed by a persuasive message (such as the quality of an expert witness’s scientific evidence) to educate and persuade those who do not naturally do so. This would help the group reach a consensus decision that is in line with a strong (rather than weak) message. On the other hand, if group discussion is dominated by group members who are heavily focused on peripheral information (such as how much money an expert was paid to testify in court), and group members are seduced by those weak arguments, deliberation might lead the group decision astray. Thus, whether deliberation will help or hinder accuracy might depend on how much discussion is focused on central, rather than peripheral, information about experts. Work on each of these situations is detailed next.

#### The value of central information in groups’ evaluation of message strength

The potential benefit of discussing central information during group decision making is clear: The more group members discuss central information within an expert’s message (e.g., the scientific evidence underlying their testimony), the more likely the group members are to evaluate the message strength accurately. Consequently, if there are dueling experts on opposite sides of an issue, and one expert’s message is stronger than the other’s, the more group members discuss the substance of the experts’ messages specifically, the more likely they are to recognize that one expert has a stronger message than the other. Group members who had the motivation and ability to have noticed the message quality discrepancy might be able to help simplify the information for those who were either unwilling or unable to do so on their own. Ultimately, this could lead to their group reaching the more “accurate” consensus verdict. For the purpose of the current study, we are defining accuracy solely as a verdict in line with scientifically strong, rather than weak, expert testimony, while acknowledging that the legal system theoretically defines a jury’s verdict as definitive and therefore *legally* accurate regardless.

The importance of discussing task-relevant information during group decision making in reaching accurate decisions has been established by several seminal studies of realistic group deliberations. For example, Kaplan and Miller’s work [24–26] demonstrates that shifts in group decisions are driven by group members sharing task-relevant information (see also [27]), particularly when the group is required to reach unanimity [26]. Yet these studies did not distinguish between task-relevant information focused on central versus peripheral information. Our study extends this work by testing whether sharing information helps groups reach better decisions when focused on central, rather than peripheral, information.

#### The value of peripheral information in groups’ evaluation of message strength

Much prior research at the individual level suggests that reliance on peripheral information during group discussion might distract groups from assessing central message strength. Yet we argue that peripheral information might actually help groups reach a unanimous decision in favor of a strong message *when that peripheral information is in the “right” hands*—that is, when the peripheral information is used by group members who are themselves already persuaded by the stronger message, to convince the others who are not. Group decision making involves a key component that individual decision making does not: intragroup persuasion. Dual-process models predict that *individuals* who process centrally (versus peripherally) are more likely to reach the correct decision on average. But for a group to reach consensus, this is only the first step. The next important step is for group members who are arguing for the substantively strong message to persuade other group members who are arguing for the weak message (that is, group members who probably did not individually rely on central information initially).

Of course, we expected that reiterating strong, central information would be effective in persuading like-minded people (i.e., other group members already prone to favor the strong message), but to reach consensus, all group members must be persuaded—including those who are either unable or unmotivated to rely on central information. In that case, the more accurate group members might need to generate arguments that are persuasive to both people who are convinced by central information *and* people who are convinced by peripheral information. Fabrigar and Petty [28] provide indirect support by demonstrating that group members sometimes need to match their appeal to what group members find appealing. In trials, what is appealing might not be what is scientifically sound. That is, arguments ideal for persuasion are clear and convincing but not necessarily well reasoned or strong [29]. Peripheral heuristics are easy to understand, persuasive, and even more convincing to group members who are not naturally inclined to assess the strength of the central information themselves. Thus, we reasoned that peripheral information might serve an important function in *group* accuracy: The more that group members who are already arguing for the strong message augment their central information with peripheral information (strategically or not), the more persuasive they might be to a group with diverse cognitive styles, ultimately facilitating group consensus on the better decision. Our study is the first test of this theory at the group level, but it is supported by HSM’s contention that systematic and heuristic processing can occur simultaneously to facilitate attitude change at the individual level (e.g., [3, 30–31]).

### Study overview and hypotheses

We conducted a study in two phases. In the first phase we assessed participants’ NFC in a large departmental mass-testing session. Based on these scores, we recruited low and high NFC participants for an ostensibly unrelated mock trial experiment several weeks later, wherein participants considered opposing plaintiff and defense expert testimony varying in argument strength. All participants heard a cross-examination of the experts focusing on peripheral information (e.g., credentials) about the experts, but half were randomly assigned to also hear central information highlighting flaws in the weaker expert’s message (e.g., quality of the research presented by the expert). We assessed the scientific accuracy of individual judgments before and after deliberation and group-level accuracy after deliberation. As discussed previously, although there is typically no ground truth to determine whether a jury’s verdict is legally “accurate” in real trials, carefully controlled experimental design enabled us to operationalize “accuracy” (for the purpose of this study) as the verdict consistent with testimony given by the expert who presented strong, rather than weak, scientific evidence (i.e., experiments with scientific integrity).

This is the first study, to our knowledge, to test the effects of NFC and central cross-examination in a realistic group setting requiring a unanimous decision, and the first to analyze the central and peripheral content of mock juror deliberation comments (but see [32] research addressing similar issues involving real jurors). The mock jury paradigm provides a realistic, engaging context for individuals to assess argument strength and reach consensus, and it has produced seminal group decision-making findings (e.g., [26, 33–34]). It also increases the practical importance of our study, providing a theoretical foundation for identifying interventions to improve jurors’ accuracy in assessing the validity of scientific evidence in court.

#### Individual-level hypotheses

Based on dual-process models, we predicted that the helpfulness of group deliberation would depend on whether participants were induced to focus on central information about the expert testimony via cross-examination (i.e., flaws in the weak expert’s message). We predicted a three-way interaction between cross-examination type, juror NFC, and deliberation: When participants are induced to focus on only *peripheral information* about an expert via cross-examination, we predicted that deliberation would make low NFC participants (who are already prone to rely on peripheral information) less accurate. In contrast, high NFC participants (who already tend to focus on central information) would be buffered against this accuracy decline during deliberation because they will naturally focus on central information even when not induced to do so via cross-examination.

In contrast, when participants are induced to focus on *central information* about an expert (in addition to peripheral information) via cross-examination, their accuracy would increase after deliberation regardless of NFC. We predicted this simple main effect in this condition because we expected that specifically highlighting flaws in the weak expert’s central message would help both high NFC participants (who are already focused on central information) and low NFC participants (who are not naturally inclined or able to rely on central information, but whom we expected to do so when prompted by cross-examination).

We also tested our theoretical explanation for this effect, predicting that when participants focus on central information about the experts’ message, deliberation would sensitize participants to the quality discrepancy between the experts’ messages, which would increase verdict accuracy.

#### Group-level hypotheses

Our group-level hypotheses were more exploratory. After coding all deliberation comments about the experts’ testimony, we calculated two proportions that represented the percentage of deliberation comments about experts that were focused on peripheral as opposed to central information (e.g., 45% would mean that 45% of a jury’s comments about the experts were peripheral and 55% were central)—we calculated a proportion at the individual-juror and jury level. We tested two competing group-level hypotheses. The first prediction was that there would be a *negative* relation between the proportion of peripheral deliberation comments and verdict accuracy, such that the more the mock jury discussed peripheral (in proportion to central) information about the expert testimony, the less likely the jury would reach more scientifically accurate verdict by siding with the expert who presented strong (versus weak) research (i.e., message strength).

The competing hypothesis is consistent with the theory outlined earlier that the most persuasive arguments need not be well reasoned, but rather must match what appeals to the listener [29]. Specifically, we predicted that there would be a *positive* relation between the proportion of peripheral deliberation comments and verdict accuracy: The more the mock jury augmented central comments with peripheral comments, the more likely the jury would agree on the scientifically accurate verdict. But, this prediction included a moderator—the scientific accuracy of the participants themselves. Specifically, we predicted that the positive relation between peripheral information discussion and a more accurate verdict would hold only when peripheral information is used by more “scientifically accurate” participants—those who already favored the expert presenting the strong message. That is, peripheral deliberation comments could make scientifically accurate participants more persuasive to their group (by appealing to what non-scientifically leaning participants like to hear), and in turn, increase the likelihood that their groups would reach consensus on the more accurate verdict. This was not predicted for scientifically inaccurate participants, because making them more persuasive would lead the group toward the *less* scientifically accurate verdict.

## Method

### Participants

Participants were undergraduate students at a large Midwestern university. Need for cognition was assessed in mass-testing sessions during three semesters from 2007–2009 (*n* = 1887 total). Participants from the top and bottom thirds of the NFC distribution (Low NFC: range = 21–56, *M* = 49.00, *SD* = 6.07; High NFC: range = 65–90, *M* = 71.32, *SD* = 5.17) were able to sign up for the ostensibly unrelated experimental sessions. Participants in this second experimental phase (*n* = 192) were assigned to 32 6-person juries (*M*_age_ = 19 years, *SD* = 1.73; 65% women; 41% White, 25% Asian, 16% Hispanic/Latino, 9% African American, 3% Native Hawaiian/Pacific Islander). NFC scores in this experimental sample (range = 34–85, *M* = 60.24, *SD* = 13.18) were similar across juries, *F*(1, 160) = .14, *p* = 1.00.

### Materials

All experimental materials and measures are included in supplemental information (see S1 File).

### Trial stimulus

The trial stimulus was an audiotaped version of a trial transcript (modified from [17]) including an actor posing as the judge who read a summary of the trial evidence and jury instructions, as well as direct-examination and cross-examination of two actors posing as expert witnesses. Participants were given a trial character sheet with the names of and facts about each of the expert witnesses and a copy of the actual Illinois pattern jury instructions appropriate for the case. Trial lawyers and an expert on jury instructions reviewed all materials to ensure their ecological validity.

The trial described a plaintiff who alleged that his colon cancer was the result of workplace exposure to chemicals called polychlorinated biphenyls (PCBs). The plaintiff’s expert witness presented a study demonstrating a link between PCBs and cancer in rats; the defense’s expert witness presented a study finding no such link. The testimony focused on this scientific evidence rather than whether the company exposed the plaintiff to the PCBs (which was made unequivocally clear in the trial summary) to ensure that verdict decisions were a pure reflection of how participants assessed the experts’ scientific testimony.

To assess participants’ evaluation of expert message strength, the transcript described two experts offering contradictory conclusions about whether PCBs are related to cancer. One expert presented a strong message; that is, testimony based on the description of a scientifically high-quality study that used valid and reliable methodology. The other expert presented a weak message; that is, testimony based on the description of a scientifically low-quality study using flawed research methodology). This allowed us to investigate how “scientifically accurate” our mock jurors and their verdicts were, by operationalizing the decisions as more accurate when they were in line with the strong (rather than weak) expert’s message. The strong study had a control group, an ecologically valid method of dosing the study rats with PCBs, and was peer reviewed and published. In contrast, the weak study did not have a control group, had a less ecologically valid method of dosing the rats with PCBs, and had not been peer reviewed or published. The complexity of the two experts’ testimonies were similar according to (a) the original study [17], (b) a Microsoft Word difficulty assessment (both testimonies = 8th grade reading level), and (c) a Linguistic Inquiry and Word Count analysis ([35]; the percentage of “big words” were similar: 18.63% of the defense testimony, 19.41% of the plaintiff testimony).

**Fig 1:**
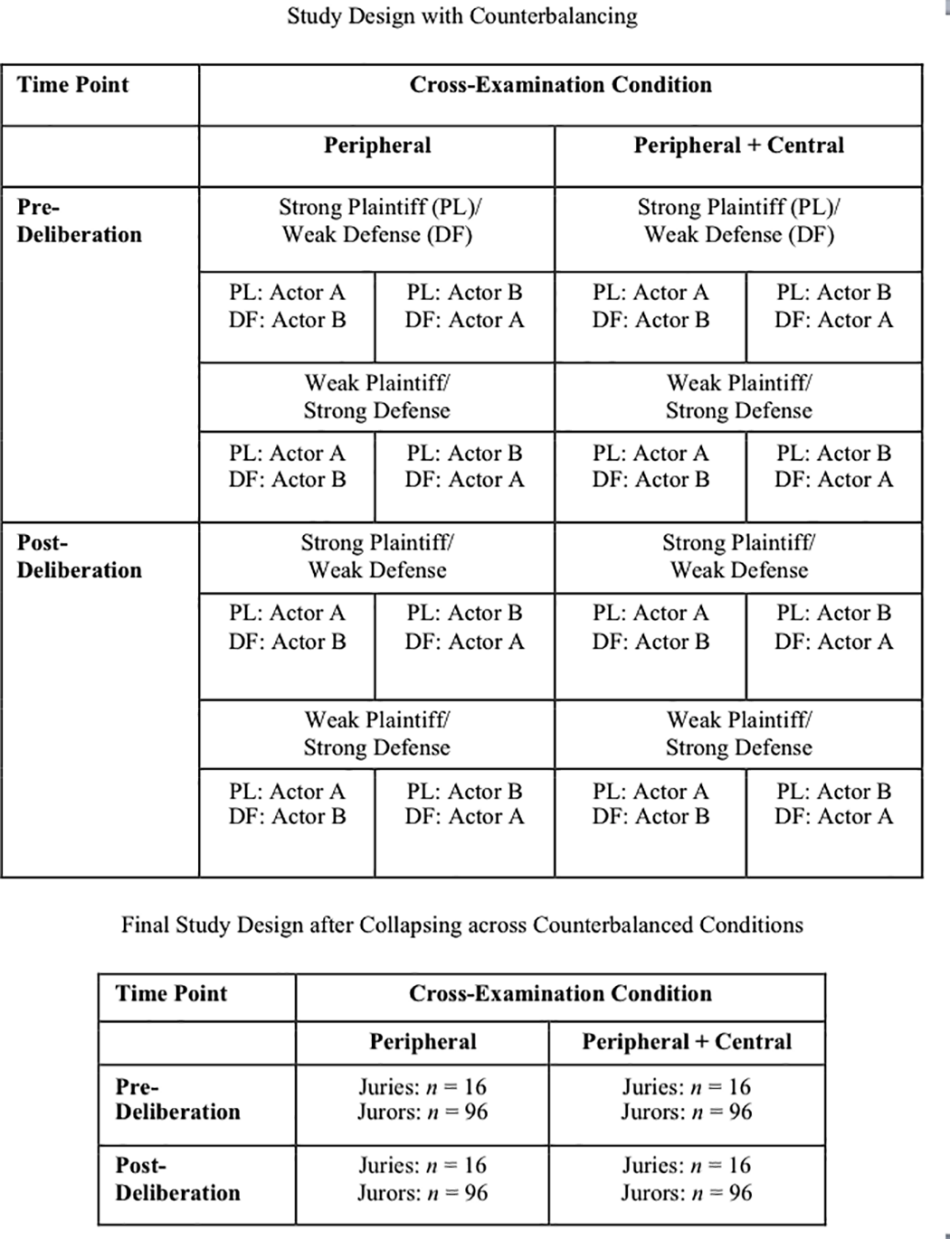
Counterbalancing conditions and final study design.

Precautions were taken to ensure that no peripheral information about the experts (e.g., their credentials, testimony record, manner of speaking, etc.) was systematically related (i.e., confounded) with their message strength. First, all peripheral information about the experts was held as constant as possible: They had similar credentials, were paid similar amounts for testifying, and testified previously in trials for their current side in this case (i.e., plaintiff vs. defense) with roughly the same frequency. Second, the audiotapes were counterbalanced, such that half of the participants heard (a) the plaintiff expert present the strong message and the defense expert present the weak message while the other half heard the reverse, and (b) Actor A as the plaintiff expert and Actor B as the defense expert while the other half heard the reverse. Therefore, peripheral information about the experts in this study was unrelated to which expert was presenting the strong versus weak message. All analyses collapsed across these counterbalanced conditions. (See Fig 1 for a diagram of the full design with counterbalanced conditions and the collapsed version used for analyses.)

### Cross-examination-type manipulation

Both experts in all conditions were cross-examined with attempts to discredit them relying on peripheral information (i.e., heuristics) that jurors sometimes use when they are unable or unmotivated to process an expert’s message: (a) their credentials and qualifications, (b) how often they testify for the plaintiff/defense, and (c) their payment for testifying (to suggest they are biased “hired guns”, [14]). This portion of the cross-examination was held constant across all conditions—both experts were always attacked via peripheral information during cross-examination. In other words, the cross-examination of the strong expert was the same across cross-examination conditions and included peripheral information similar to the peripheral cross-examination of the weak expert.

We manipulated whether—in addition to this peripheral cross-examination information—participants also heard a portion of the cross-examination that drew attention to central information that highlighted flaws in the weak expert’s message (i.e., the lack of control group, non-ecologically valid method of dosing the rats, and lack of peer review). Thus, all participants heard attacks on the weak expert via peripheral information, but in addition, half of the participants also heard the weak expert attacked via central information about flaws in the expert’s message itself (“central + peripheral”). The other half did not (“peripheral only”). We chose this manipulation, rather than a purely peripheral versus purely central cross-examination, to test whether focusing cross-examination on central information can improve mock jurors’ assessment of experts above and beyond the standard peripheral information that attorneys are taught to use (e.g., [36–37]).

### Measures

#### Need for cognition [38]

Participants completed the 18-item short form of the NFC scale (α = .85). This scale assesses a person’s intrinsic motivation to process information thoroughly (e.g., “I would prefer a task that is intellectual, difficult, and important to one that is somewhat important but does not require much thought”) on a 5-point response scale ranging from 1 (*Extremely Uncharacteristic*) to 5 (*Extremely Characteristic*). NFC is an oft-used and valid predictor of an individual’s natural inclination to engage in central processing [6].

#### Individual verdict accuracy

**Fig 2:**
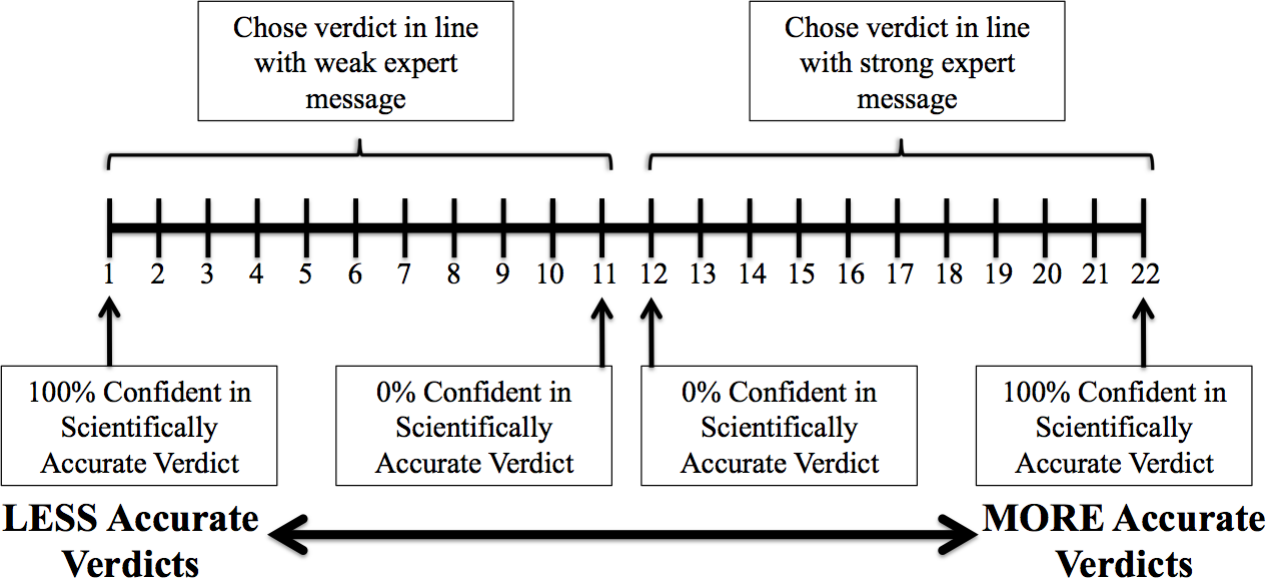
Individual verdict accuracy scale.

After the evidence presentation, but before deliberation, participants individually chose pre-deliberation verdicts, which were recoded to reflect whether the participants chose the “scientifically accurate” or “scientifically inaccurate” verdict (i.e., the verdict in line with the strong or weak expert message, respectively). This coding depended on the counterbalancing condition: If the participants heard a strong plaintiff and weak defense expert, the pro-plaintiff verdict would be the “scientifically accurate” verdict, and vice versa. Participants also indicated confidence in their verdict on an 11-point scale ranging from 0% to 100%. This measure was combined with their verdict to create a more sensitive 22-point *individual verdict accuracy* bipolar scale ranging from 100% confident in the scientifically accurate verdict to 100% confident in the scientifically inaccurate verdict (Fig 2; e.g., [39–40]).

#### Group verdict accuracy

A dichotomous variable reflected whether the group reached consensus on the scientifically accurate verdict or was unable to reach consensus on the scientifically accurate verdict (either because they reached consensus on the scientifically inaccurate verdict or because they were unable to reach consensus at all).

#### Expert credibility

Participants indicated their perceptions of the plaintiff and defense experts’ credibility, based on the evidence they presented, on a 6-point scale ranging from *Very Not Credible* to *Very Credible* (e.g., [41–42]) before and after deliberation.

#### Participants’ persuasiveness to their co-jurors

Participants rated how persuasive each of their co-jurors had been during deliberation on a 5-point scale from *Not at all* to *Very*. Each participant’s rating score was the average of their 5 co-jurors’ ratings of their persuasiveness.

#### Demographics

Gender, age, ethnicity, and U.S. citizenship were assessed.

### Procedure

In Phase 1, participants completed the NFC scale during mass testing sessions, where nearly the entire university subject pool simultaneously completed numerous and varied measures. Several weeks to three months later, participants in the top and bottom third of the NFC distribution were allowed to sign up for Phase 2, the ostensibly unrelated mock trial experiment. To obtain 3 high- and 3 low-NFC participants for each Phase 2 mock trial session, all sessions had two separate sign-up postings that each included four available participant spots: one posting visible to high NFC participants and one posting visible to low NFC participants (without participants’ knowing that NFC was the selection criterion). We overbooked in case of no-shows to ensure 3 high and 3 low NFC participants on each mock jury. If more than 3 of each showed up for a session, we randomly excused extra participants.

After providing written consent, participants reviewed the trial character sheet (which they kept throughout the study for reference) and listened to the audio recording of the judge’s summary of trial evidence, while reading the transcript of this audio recording. Next, they listened to one of the eight audiotaped versions of the expert testimony (encompassing the cross-examination-type manipulation and the expert quality and expert actor counterbalanced conditions) and then they listened to Illinois Pattern jury instructions. Next, participants completed individual pre-deliberation verdict and expert credibility measures, after which they deliberated with their group until a unanimous group verdict was reached, or until half an hour had elapsed (20% did not reach consensus in that time). All deliberations were videotaped. After deliberation ended, participants individually completed post-deliberation verdicts, confidence, expert credibility, and participant persuasiveness measures. Finally, participants were thanked, debriefed, and given course credit for their participation. Each session took approximately 2 hours. Deliberations were transcribed. All materials and procedures were approved by the University of Illinois at Chicago Institutional Review Board.

### Deliberation coding

We developed a novel coding protocol to evaluate the extent to which participants relied on central versus peripheral information when attempting to reach consensus; specifically, whether participants were discussing the expert’s central message or peripheral information about the expert’s credibility as a source when deciding which expert to trust. Two independent coders, blind to condition, reliably coded 20% of the transcripts according to the criteria described below. Disagreements were resolved through discussion and each rater coded half of the remaining transcripts.

The coding protocol first established “deliberation comment” units (i.e., the smallest meaningful unit of information; 94% agreement), which were then coded for mentions of at least one expert (90% agreement). Of the 3959 deliberation comments, 41% (*n* = 1642) were about at least one of the expert witnesses (21% about plaintiff expert, 13% about defense expert, 7% about both experts). Each expert-related comment was further coded for evidence of reliance on central or peripheral information, rejection of peripheral information, or unclassifiable statements (over 86% agreement on all codes).

#### Central deliberation comments

Comments in which participants brought up the substantive content of the expert’s message, such as his study methodology, were coded as indicative of reliance on *central information*. For example, “Dr. Fallon didn’t have a control group and he injected the rats with larger doses, it wasn’t the doses the employees were exposed to.” In this comment, the participant is reviewing the message content and evaluating its merit.

#### Peripheral deliberation comments

Comments in which participants brought up a heuristic about the expert as a source (e.g., payment for testifying) that did not focus on the substantive message of his testimony, were coded as indicative of reliance on *peripheral information*. For example, “Dr. Campbell testified more for the companies, they can, like, compensate him more than the plaintiff could” [sic]. In this comment, the participant is arguing to not rely on Dr. Campbell’s testimony because he testified for a company that could pay him more than a single plaintiff could him.

#### Rejection-of-peripheral deliberation comments

Rejection-of-peripheral deliberation comments were comments in which participants *explicitly* rejected peripheral information as an unreliable basis for judging expert credibility. For example, “I wasn’t sure why they brought the whole ‘getting paid for’ either, I found it irrelevant… like they both got paid for it anyways.” In this comment, the participant is arguing against relying on the peripheral information that the expert got paid to judge credibility because it applies to both experts.

#### Unclassifiable comments

Finally, comments were coded as *unclassifiable* when it was not clear whether the participant’s statement was based on reliance on central or peripheral information. For example, “I just believed the first doctor so much more.” In this comment, it is unclear whether the participant believes the doctor more because of central information (e.g., his research) or peripheral information (e.g., where he went to school).

## Results

### Individual verdict accuracy

Descriptive statistics for all participants’ case judgments are reported in Table 1. Our first analyses tested our hypothesis that the helpfulness of deliberation would depend on participants’ NFC and whether cross-examination focused participants’ attention on the expert’s central information. We tested the predicted 3-way interaction with a 3-level multilevel model that nested repeated measures within individual participants, and participants nested within juries. The model accounted for the non-independence of pre- versus post-deliberation judgments by clustering these judgments within individual participants. That is, the same participant rendered the pre- and post-deliberation judgments. The model also accounted for the non-independence of individual judgments by clustering all participants’ judgments within their respective jury. That is, each jury comprised 6 participants, making each grouping of 6 people share an experience different from each other grouping of 6. Our model accounted for this extraneous variance. The model included the following predictors: (a) the effects of pre-deliberation versus post-deliberation judgments, within individuals (Level 1), (b) participants’ continuous NFC scores, between individuals (Level 2), (c) cross-examination type, between groups (Level 3), and (d) all possible interactions on individual verdict accuracy (controlling for the counterbalanced witness-quality version).

**Table 1:** 

	Overall	Central + Peripheral Cross-Examination		Peripheral Cross-Examination	
		Pre-Deliberation	Post-Deliberation	Pre-Deliberation	Post-Deliberation
Individual verdict accuracy	16.39	16.28	17.51	15.27	16.52
	(7.03)	(6.54)	(6.87)	(7.12)	(7.43)
Credibility of strong expert	4.83	4.52	5.07	4.64	4.76
	(0.98)	(1.59)	(0.98)	(0.98)	(0.96)
Credibility of weak expert	4.37	4.45	3.52	4.88	4.36
	(1.25)	(1.64)	(1.25)	(1.06)	(1.20)

Means (SDs) for Case Judgments as a Function of Cross-Examination Type and Deliberation

*Notes*. Individual verdict accuracy ranges from 1 (*100% confident in incorrect verdict*) to 22 (*100% confident in correct verdict*). Expert credibility scores range from 1 (*Very not credible*) to 6 (*Very credible*). As noted in methods, “Peripheral Cross-Examination” refers to a cross-examination of the weak expert that focused exclusively on peripheral information against the expert. “Central + Peripheral Cross-Examination” refers to a cross-examination of the weak expert that included both the peripheral information but also central information against the expert.

**Fig 3:**
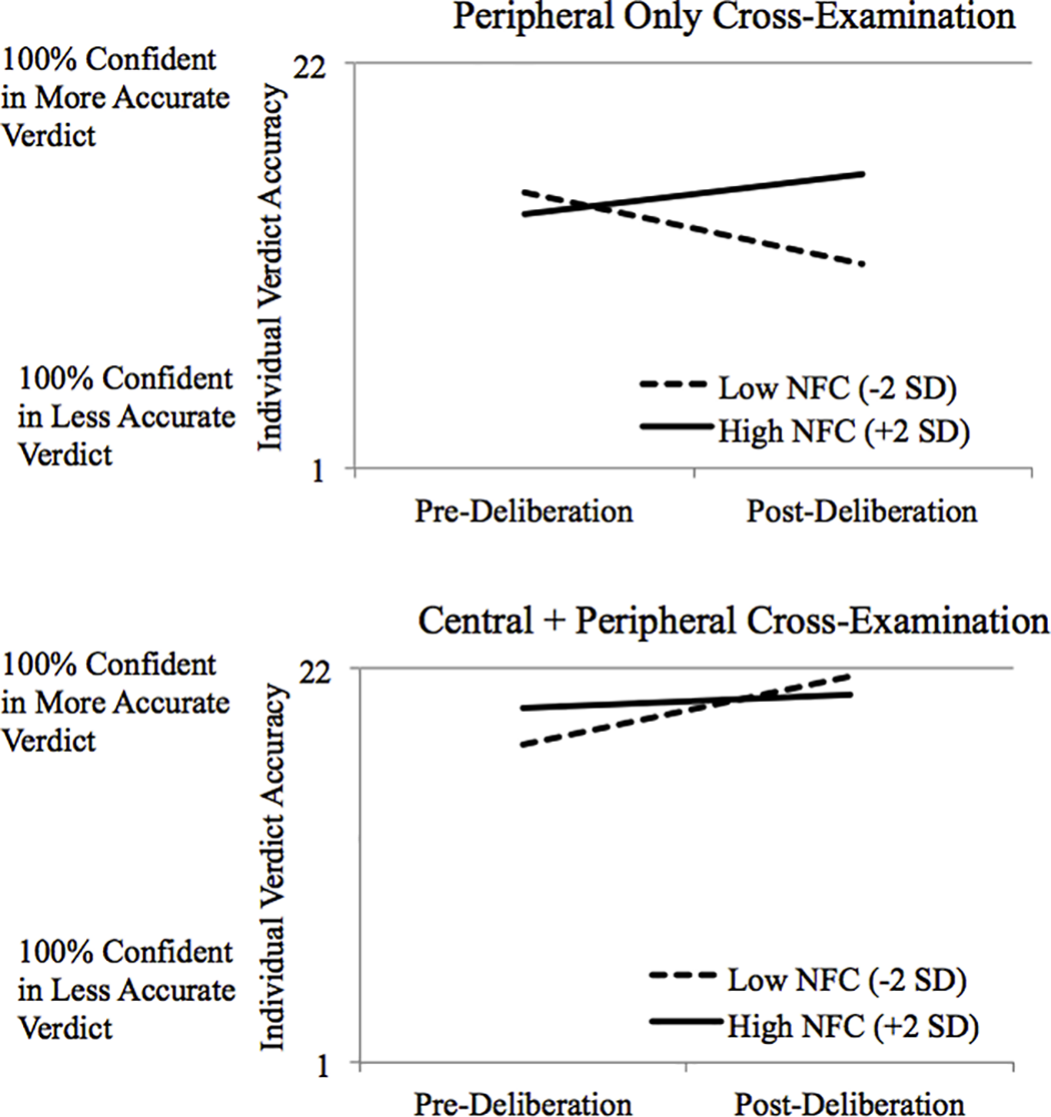
Individual verdict accuracy as a function of pre- versus post-deliberation, NFC, and cross-examination type condition.

Individual participants were more confident in the accurate verdict when they heard a cross-examination that focused them on central (versus peripheral) information about the expert, *B* = 4.17, *t*(28) = 3.53, *p* = .002, 95% *CI*s = [1.85, 6.49]. This effect (and a significant deliberation × NFC interaction, *B* = -.11, *t*[365] = 3.00, *p* = .003, 95% *CI*s = [.04, .19]) were qualified, however, by the predicted significant three-way interaction, *B* = -.17, *t*(365) = -3.18, *p* = .002, 95% *CIs* = [-.27, -.06] (see Fig 3). No other main effects or two-way interactions were significant, *B*s ≤ |2.99|, *p*s ≥ .130.

To probe this interaction, we assessed the simple deliberation x NFC interaction separately for each cross-examination type condition. As expected, when participants heard the cross examination that focused on *central information* against the weak expert, group deliberation made them more accurate, *B* **=** 2.17, *t*(365) = 6.05, *p* < .001, 95% *CI*s = [1.46, 2.87]—regardless of their need for cognition. That is, the simple deliberation × NFC interaction was not significant in this condition, *B* = -.05, *t*(365) = -1.50, *p =* .13, 95% *CI*s = [-.13, .02]. In contrast, when participants heard the cross examination that included *only* peripheral information, we found a significant simple deliberation × NFC interaction, *B* = .11, *t*(365) = 3.00, *p* = .003, 95% *CI*s = [.04, .18]: Lower NFC participants (i.e., who are dispositionally prone to rely on peripheral information) became marginally *less* accurate over the course of deliberation, *B* = -3.77, *t*(365) = -1.90, *p* = .058, 95% *CI*s = [-7.68, .14], 90% *CI*s = [-7.05, -.49], but higher NFC participants were buffered against this decline in accuracy during deliberation, *B* = 2.12, *t*(365) = 2.34, *p =* .37, 95% *CI*s = [-2.49, 6.73.]

#### Mediation analysis: Why did deliberation increase accuracy when guided by cross-examination that focused on central information?

**Fig 4:**
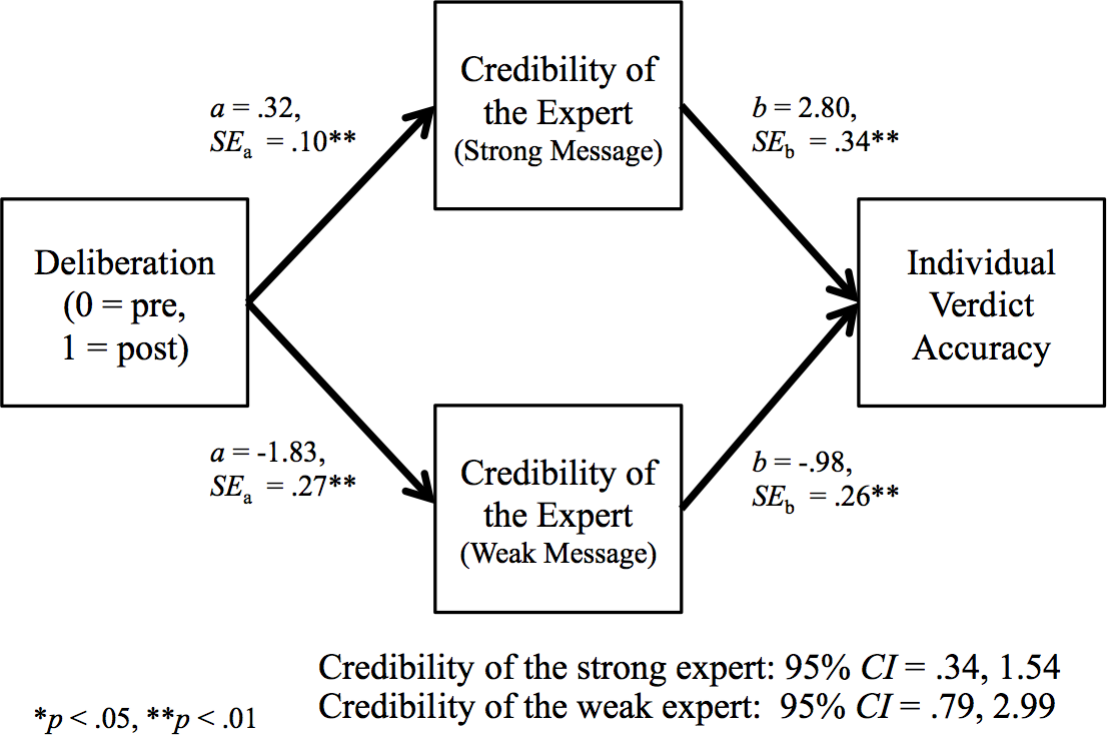
The indirect effect of deliberation on individual verdict accuracy through expert credibility.

At the individual level, we expected that (consistent with dual-process models), when deliberation was guided by a cross-examination that focused participants on central information against the weak expert, they would be sensitized to the quality discrepancy between the two experts as a result of group deliberation (i.e., from pre- to post-deliberation), and in turn their individual verdict accuracy would increase after deliberation. Because the analyses described above revealed that deliberation helped (i.e., made individual participants more accurate) only when they had been focused on central information about the weak expert during cross-examination, we limited the mediation analysis to this condition. We followed Selig and Preacher’s [43] instructions for Monte Carlo simulations to assess mediation with their online tool for generating indirect-effects confidence intervals (http://www.quantpsy.org/medmc/medmc.htm). This analysis included the path coefficients and associated standard errors from (a) two multilevel linear regressions (with pre- and post-deliberation judgments nested within individual participants and participants nested within juries), each with the independent variable (deliberation) predicting one of the two mediators (perceived credibility of the expert presenting strong and the expert presenting weak messages; i.e., *a* paths in Fig 4), and (b) one multi-level linear regression with the perceived expert credibility measures predicting the dependent variable (individual verdict accuracy; i.e., *b* paths in Fig 4) with the independent variable (deliberation) included.

The dual-mediator model analysis tested the indirect effects of deliberation on individual verdict accuracy through participants’ assessments of the credibility of the experts in the central + peripheral cross-examination condition.

This analysis revealed that the predicted indirect effects of deliberation on individual participants’ verdict accuracy through perceptions of both the strong and weak experts’ credibility were significant—but with very different patterns. Deliberation made participants rate the expert presenting the strong message as more credible, which in turn made them more confident in the scientifically accurate verdict. Deliberation also made participants rate the expert presenting the weak message as less credible, which in turn also made them more confident in the scientifically accurate verdict. Thus, as predicted—and consistent with dual-process models (i.e., ELM, HSM)—when individuals were focused on central information about the weak expert, group discussion sensitized them to differences in the experts’ message strength, which in turn made them more confident in the scientifically accurate verdict—without making them skeptical of both experts.

In summary, factors promoting reliance on central information (i.e., NFC, cross-examination based on central information) about the weak expert made individuals within groups more confident in the verdict that was consistent with the stronger expert. Specifically, highlighting flaws in the weak message made mock jurors more confident in the scientifically accurate verdict, overall. Hearing a cross-examination that focused mock jurors on flaws in the expert’s central message also made deliberation more helpful by sensitizing them to the quality discrepancy between the two experts. Deliberation was not helpful, however, when the cross-examination focused exclusively on peripheral information about the experts. In this condition, low NFC participants (who were naturally inclined to process peripherally) became even less accurate, while high NFC jurors were buffered against this decline in accuracy during deliberation.

### Group accuracy results

The 32 juries were coded as 0 for failing to reach consensus on the scientifically accurate verdict (i.e., unanimously agreeing on the verdict consistent with the weak expert or failing to reach consensus, *n* = 19, 60%) or as 1 for reaching consensus on the scientifically accurate verdict (*n* = 13; 40%). Given that we collapsed across counterbalancing conditions, this allowed for 16 groups and 96 participants in each of the two cross-examination-type conditions. It is important to note that participants spent most of their time discussing the experts’ message. That is, the majority of all comments about experts were focused on central information (80%, see Table 2). When participants *did* make peripheral deliberation comments (14%), they were most commonly referring to the experts’ credentials and how often they testified for the prosecution or defense, but also the expert’s payment for testifying and manner of speaking while testifying (Table 2). On the rare occasion (3%) that participants made a comment explicitly rejecting peripheral information, they were typically rejecting the expert’s payment as a basis for determining credibility.

**Table 2:** Breakdown of comments made about experts during deliberation (n = 1642).

Comment Type	*N*	%
**Central Deliberation Comments**	**1312**	**80%**
**Peripheral Deliberation Comments**	**229**	**14%**
Expert’s credentials	71	31%
How often the expert testified for prosecution vs. defense	66	29%
The expert’s payment for testifying	31	13.5%
The expert’s personality or manner/style of speaking	30	13%
Speculation about other ulterior motives for testifying	10	4%
Procedural factor (e.g., the fact that there were two opposing experts)	10	4%
Other	11	5%
**Rejection-of-Peripheral Deliberation Comments**	**41**	**3%**
The expert’s payment for testifying	27	66%
How often the expert testified for prosecution vs. defense	9	22%
Expert’s credentials	3	7%
Speculation about other ulterior motives for testifying	2	5%
**Unclassifiable Comments**	**56**	**3%**
Comment Type	*N*	%
**Central Deliberation Comments**	**1312**	**80%**
**Peripheral Deliberation Comments**	**229**	**14%**
Expert’s credentials	71	31%
How often the expert testified for prosecution vs. defense	66	29%
The expert’s payment for testifying	31	13.5%
The expert’s personality or manner/style of speaking	30	13%
Speculation about other ulterior motives for testifying	10	4%
Procedural factor (e.g., the fact that there were two opposing experts)	10	4%
Other	11	5%
**Rejection-of-Peripheral Deliberation Comments**	**41**	**3%**
The expert’s payment for testifying	27	66%
How often the expert testified for prosecution vs. defense	9	22%
Expert’s credentials	3	7%
Speculation about other ulterior motives for testifying	2	5%
**Unclassifiable Comments**	**56**	**3%**

We created a proportion to represent the extent to which mock jurors and juries relied on peripheral, rather than central, information during deliberation. Because rejection-of-peripheral deliberation comments were so rare (3%) and more conceptually similar to central comments, we combined them with central deliberation comments. To compute the “proportion of peripheral deliberation comments” for each jury, we divided the number of peripheral deliberation comments by the number of all classifiable comments about the experts. That is, mathematically: peripheral deliberation comments / (peripheral + central + reject-peripheral deliberation comments) for each jury. We also calculated this proportion for each individual participant. For example, if Participant A made 10 peripheral comments, 55 central deliberation arguments, and 5 reject-peripheral deliberation arguments, her proportion of peripheral comments would be 14%. This would be interpreted to mean that this mock juror relied on peripheral information 14% of the time (and, conversely, she relied on central or rejection-of-peripheral information 86% of the time). If Participant B had a higher proportion of peripheral comments (e.g., 20%), he spent more time discussing peripheral, rather than central, information relative to Participant A.

#### Do mock juries’ peripheral deliberation comments make them more or less likely to reach the scientifically accurate verdict?

Next, we tested competing hypotheses regarding whether discussing more peripheral information about the conflicting expert messages would make mock juries more or less likely to side with the strong, rather than weak, expert message. Our logistic regression with jury-level proportion of peripheral (versus central) deliberation comments predicting jury-level verdict accuracy revealed that the more a jury discussed *peripheral information* about the expert, the more likely it was to reach the scientifically accurate verdict (i.e., the verdict in line with the strong expert message), *B* = 6.19, Wald = 3.91, *p* = .048, 95% *CI*s = [-.22, 12.61], 90% *CI*s = [.86, 11.53]. It is again important to note that this is in the context of a high baseline of central deliberation comments (80%). Thus, our results do not suggest that peripheral information on its own improves decision quality. Rather, it is the use of central information, augmented by the use of some peripheral information, that led mock juries to reach consensus on the scientifically accurate verdict.

#### Do scientifically accurate (but not scientifically inaccurate) participants’ peripheral deliberation comments increase group accuracy because they are more persuasive?

Our next analyses tested our explanation for this effect: Peripheral deliberation comments would make scientifically accurate participants more persuasive and thereby more likely to convince their mock jury of the more accurate decision. This was not predicted for scientifically inaccurate participants, because making them more persuasive would lead people toward the *less* accurate verdict.

We conducted a multilevel logistic regression with pre- and post-deliberation judgments nested within individual participants, and participants nested within juries. The following predictors were included: (a) individual- participant-level proportion of peripheral (rather than central) deliberation comments and a dummy code reflecting whether the participant preferred the scientifically accurate (0) or scientifically inaccurate (1) verdict before deliberation (Level 1), (b) cross-examination type (Level 2), and (c) all interactions predicting whether their jury reached the more accurate verdict.

**Fig 5:**
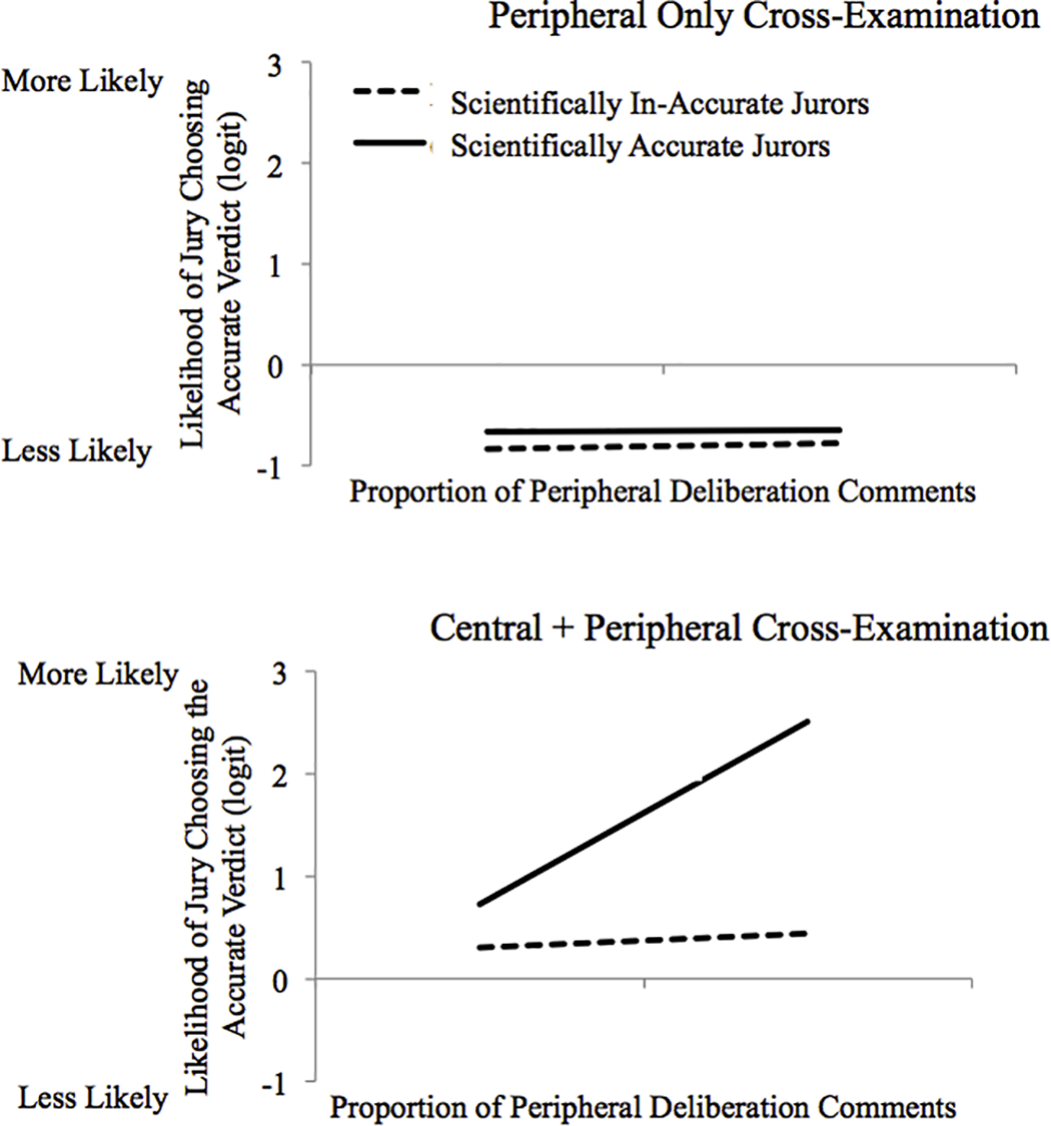
The likelihood of the jury choosing the more accurate verdict as a function of arguments made during deliberation.

A significant three-way interaction, OR = 5.49, *t*(114) = 3.42, *p* = .001, 95% *CI*s = [.72, 2.68], indicated that when cross-examination focused participants on *central information*, the effectiveness of peripheral deliberation comments depended on whether they were “in the right hands”; that is, whether they were used to augment the comments of participants who were arguing for the scientifically accurate or scientifically inaccurate verdict. To probe the interaction, we assessed the simple interactions between peripheral deliberation comments and whether the participant was arguing for the scientifically accurate verdict separately for each cross-examination-type condition. This simple interaction was significant when the cross-examination highlighted flaws in the expert’s central message, OR = 5.22, *t*(114) = 3.41, *p* = .001, 95% *CI*s = [.70, 2.61] (Fig 5). More specifically (and as predicted), simple slopes revealed that scientifically inaccurate participants’ proportion of peripheral deliberation comments did not increase their jury’s accuracy, OR = 1.15, *t*(114) = .42, *p* = .67, 95% *CI*s = [-.51, .79]. Yet, scientifically *accurate* participants’ proportion of peripheral deliberation comments positively predicted their jury’s accuracy, OR = 6.01, *t*(114) = 4.43, *p* < .001, 95% *CI*s = [.99, 2.59]. In other words, the more that participants arguing for the scientifically accurate verdict used peripheral information (in addition to their high baseline of central information), the more likely was their mock jury to reach the more scientifically accurate verdict.

The three-way interactive effect of whether the participant favored the scientifically accurate or non-accurate verdict, the proportion of peripheral deliberation comments, and cross-examination type condition on the likelihood of the jury choosing the more accurate verdict.

In contrast, when participants were focused on only *peripheral information* about the weak expert via cross-examination, the simple interaction between whether participants were scientifically accurate and their proportion of peripheral deliberation comments was not significant, OR = .95, *t*(114) = -.45, *p* = .65, 95% *CI*s = [-.27, .17]. The mock jury’s accuracy remained low in this condition regardless of how much peripheral information participants discussed and whether they were arguing for a scientifically accurate or non-accurate verdict—these simple main effects were not significant, *B*s ≤ .17, *p*s ≥ .12.

#### Mediation analyses: Why did scientifically accurate participants’ peripheral deliberation comments increase accuracy?

Next we tested whether this effect was due to scientifically accurate (but not scientifically inaccurate) participants being more persuasive when they brought up peripheral information during deliberation. First, we repeated the linear multilevel regression model, with participants’ proportion of peripheral deliberation comments and a dummy code reflecting whether the participants preferred the scientifically accurate (0) or scientifically inaccurate (1) verdict pre-deliberation (Level 1), (b) cross-examination type (Level 2), and (c) all interactions as predictors of participants’ persuasiveness to their co-jurors during deliberation.

**Fig 6:**
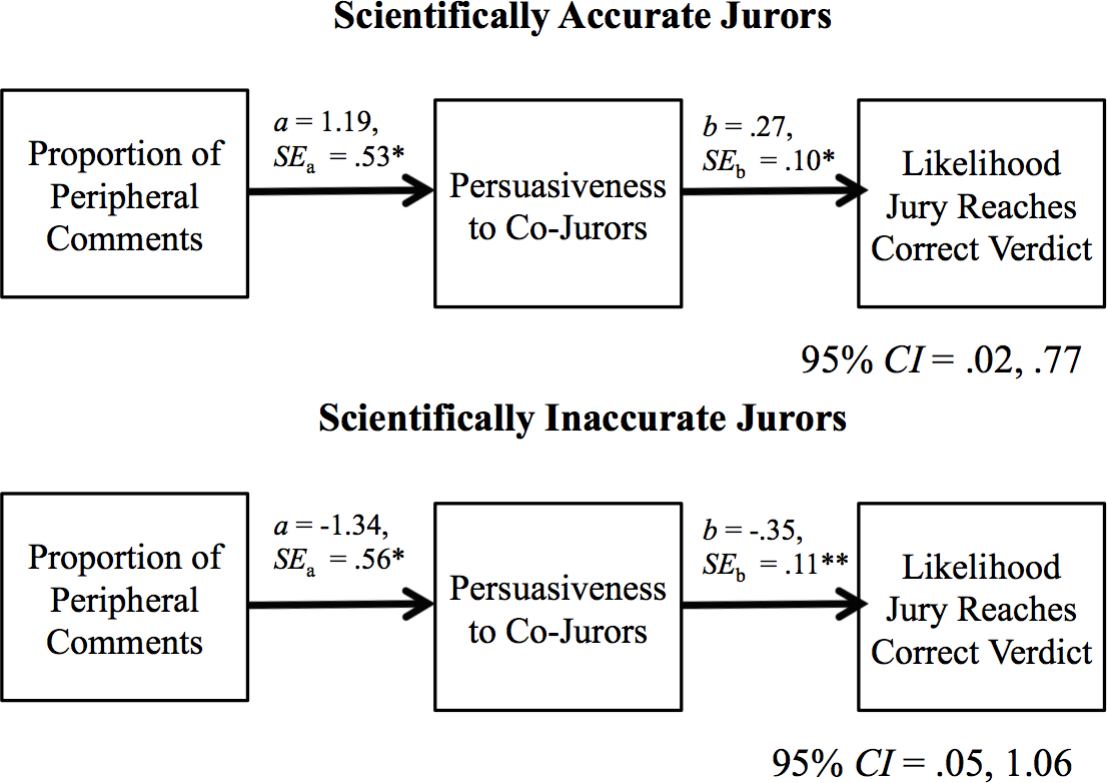
The indirect effect of peripheral comments on scientifically accurate verdicts through individuals’ persuasiveness.

Because scientifically accurate participants’ peripheral deliberation comments made their juries more accurate *only* when the cross-examination highlighted flaws in the weak expert’s central message, we limited the mediation analysis to this condition. We conducted a Monte Carlo simulation [43] including the path coefficients and associated standard errors from (a) a multilevel linear regression (with individual participants nested within juries) with the independent variable (peripheral deliberation comments) predicting the mediator (perceived persuasiveness; i.e., path *a* in Fig 6), and (b) a multilevel logistic regression with the mediator (perceived persuasiveness) predicting the dependent variable (jury accuracy) with the independent variable included (i.e., path *b* in Fig 6).

The indirect effect of the proportion of peripheral deliberation comments on the group’s likelihood of reaching the scientifically accurate verdict through participants’ persuasiveness to their co-jurors as a function of whether the participant was scientifically accurate or non-accurate (in central + peripheral cross-examination condition only).

The indirect effect of participants’ peripheral deliberation comments on their group’s accuracy through perceptions of their persuasiveness was significant for both scientifically accurate and scientifically inaccurate participants—but with very different patterns. As predicted, the more that scientifically accurate participants made peripheral deliberation comments, the more persuasive they were to their co-jurors, and in turn, the more likely their mock jury was to reach the more scientifically accurate verdict. In contrast, both paths were negative for scientifically inaccurate participants: The more they used peripheral deliberation comments, the *less* persuasive they were; also, the more persuasive they were, the *less* likely their group was to reach the more accurate verdict. This significant indirect effect for scientifically inaccurate participants suggests that their use of peripheral deliberation comments also increased group-level accuracy–yet for a different reason than for scientifically accurate participants: Peripheral deliberation comments *reduced* scientifically inaccurate participants’ persuasiveness, which otherwise would have led the jury toward the less accurate verdict.

#### Raw count of deliberation comments predicting group accuracy

For the previous analyses, we had calculated proportions (rather than analyzing the raw number of comments) because proportions are the best method for capturing the theoretical construct of interest (i.e., the extent to which deliberation comments were peripheral versus central), while controlling for the raw number of deliberation comments. Even so, one might wonder (as an anonymous reviewer did), whether juries who deliberated longer (i.e., made more comments) were more likely to agree on the more scientifically accurate verdict. To address this question, we ran a post-hoc and exploratory logistic regression with the raw number of comments that each jury made predicting whether the group reached the more scientifically accurate verdict. Not only did increasing the number of comments not significantly increase the group’s likelihood of reaching the correct verdict, we found an unexpected effect in the reverse direction: The more comments that the group made, the less likely the group was to reach the correct verdict, *B* = -.01, Wald = 4.85, *p* = .033, 95% *CI* = [-.04, -.004]. It is important to note, however, that this effect is significant only when using an uncorrected *p*-value threshold for significance (*p* < .05) as though we were conducting an analysis to test for an effect we had predicted, which we were not. Thus, the juries’ accuracy was driven not by the raw number of comments that were made, but instead specifically by the proportion of those comments that were peripheral comments about the experts.

## Discussion

Despite the pervasiveness of group-based persuasion in everyday life, studies directly testing dual-process predictions for group-level persuasion where the group’s members have to deliberate together to reach consensus are rare. We examined the mechanisms and outcomes of persuasive communication at the individual and group-level simultaneously by employing a carefully controlled experimental design and advanced methodological and statistical tools. This enabled a novel test of dual-process models (ELM, HSM) in the context of group decision making, where most prior work focused on individual-level decision making. Our work, using mock trial methodology, makes contributions to the social psychological literature on decision making but also to the field of psychology and law, where jury decision making is of great theoretical and applied interest.

Consistent with dual-process models, *individuals* evaluated argument strength more accurately when intrinsically (determined by their level of need for cognition) and/or extrinsically (determined by cross-examination focus) motivated to focus on central or substantive information within the testimony of an expert witness. Specifically, when cross-examination focused individuals on an expert’s central message (i.e., the scientific quality of the studies he presented to support his conclusions), group discussion during deliberation sensitized them to a substantive credibility discrepancy between experts. In turn, individuals were more confident in the strong expert’s argument, and led them to favor a more scientifically accurate verdict. In contrast, when cross-examination focused individuals on peripheral heuristics about experts (e.g., credentials, payment for testifying), group deliberation made some individuals favor a verdict that was not consistent with the scientific evidence presented. Specifically, low NFC individuals (who naturally find peripheral information persuasive) were less accurate after deliberation, but high NFC individuals (who are not predisposed to being persuaded by peripheral information) were buffered against this decline in accuracy during deliberation.

At the level of group discussion, we discovered that participants were most persuasive to other mock jurors during deliberation when they supplemented central information with peripheral information. This group process has not before been described in the scientific literature. Detailed coding of deliberation comments revealed that the more groups discussed peripheral information about the experts that was completely *unrelated* to message quality (how much he was paid, etc.), the more likely they were to reach the accurate decision in line with the strong expert who presented a high-quality study. But this occurred only when the peripheral information was used to bolster arguments being made by the participants who were already favoring the scientifically accurate verdict. Therein lies the value of peripheral information: It makes the participants who are arguing for the more accurate decision more persuasive to people who might have been either unable or unmotivated to rely on central information about the expert.

Although it would not be particularly surprising that peripheral information is persuasive in general, this study demonstrates that peripheral information can help groups make decisions that reflect more accurate assessments of central argument strength—even when the peripheral information is intentionally unrelated to argument strength. Our study is unique given the complexity of the methodological precautions used to ensure that peripheral information was unrelated to expert argument strength, which enabled us to demonstrate that unrelated peripheral information can help groups, specifically, reach better assessments of message strength. This finding suggests that the process through which individuals reach accurate assessments of argument strength in the aggregate gets more complicated at the group level given the diversity of thinking styles and opinions in a group whose members must reach consensus. The value of peripheral information about experts in our study is that it made participants who are prone to recognize flaws in a central comments more persuasive to others who either cannot grasp the implication of central information or simply do not find it compelling, which ultimately led to their group being more likely to agree on the more accurate decision in line with the strong expert.

One interesting question raised by our data is whether this is a conscious strategy to convince others. Do scientifically accurate participants, who were convinced to side with the strong expert because of central information, attempt to use only central information, but when they realize they are getting nowhere with that strategy, do they switch to peripheral information (that they might not have themselves found compelling) just to convince others? Do they intuitively “know their audience”—do they sense that some people are more likely to be convinced by less central details, by heuristic processing? This is a fascinating question that we believe would be very fruitful for future research, but given that our study was not designed to test it, we remain agnostic at this point. Our data speak only to the argument that the more scientifically accurate participants happen to make peripheral comments about the experts—strategically or by chance—the more likely they are to get their group to agree on the verdict in line with the strong expert. We hope this finding will inspire future researchers to investigate more deeply the mechanism for this effect; for example, whether it is due to people matching peripheral information to people who find it compelling when central information fails [28], due to peripheral information bolstering the source credibility of a message participants already found persuasive [44]—potentially in a post hoc rationalization process, or due to other ways in which peripheral information made scientifically accurate participants more persuasive to the group.

### The value of combining central and peripheral information during group discussion

What advice would we give group members trying to bring their group to the accurate decision? Should they use central or peripheral information? Groups are diverse and will surely be composed of those who find central information compelling, but also those who find peripheral information compelling. Our results suggest that *both* types of information are necessary for several reasons. First, the baseline use of central information during deliberation was very high: On average, groups discussed central information about the experts’ testimony 80% of the time. Thus, our results should not be erroneously interpreted as an argument in favor of using peripheral information *instead* of central information; rather, we suggest that using at least some peripheral information above and beyond a large base of central information will appeal to groups of diverse opinions and might be more likely to bring a group to the more accurate decision than would focusing exclusively on central information.

Second, we found that peripheral information helped scientifically accurate participants convince their co-jurors of the more accurate decision only when deliberation followed exposure to a version of cross-examination that included central information about the expert’s message quality. Thus, for scientifically accurate participants to sway others with peripheral information, they (a) must themselves be certain that their opinion is correct, perhaps via cross-examination that clearly highlighted the flaws in the weak expert’s study, and (b) need an arsenal of central information in conjunction with the persuasive peripheral information. Without the central cross-examination focusing on central information against the weak expert, there might not have been a critical mass of participants who had confidently reached the scientifically accurate verdict before deliberation in the first place, and those who *had* might not have had enough of a foundation of central information upon which the peripheral information could be successfully added to build a more persuasive message.

### Theoretical implications

The current work has important theoretical implications. It has been argued that group processes can differ quantitatively from individual processes by reproducing, amplifying, or attenuating individual processes [45–46], suggesting that processes are similar for individuals and groups but differ in degree. Although differences between individual and group outcomes can often be explained by similar processes, there are some exceptions [47]. For example, Wright, Luus, and Christie [48] found that groups were sensitive to consensus information but individuals were not, which suggests a different process. Our data tentatively suggest another way that group processes might differ qualitatively from individual processes. Although peripheral information can be detrimental to individuals’ assessment of argument strength, peripheral information about a message source (even if irrelevant to the source’s argument strength by design) can play a helpful role in during group discussion. Peripheral heuristics about a source can be beneficial to *groups* assessing argument strength not because they are actually diagnostic of argument strength, but because they help jurors arguing for the more accurate assessment of argument strength be more persuasive, which is required for the group to reach consensus. Thus, dual-process theories need to account for the cognitive diversity of people who need be convinced to achieve consensus—including those who are either unable or unwilling to rely on strong central information.

The fact that peripheral information helped groups evaluate argument strength when used in combination with central information is consistent with the Heuristic-Systematic Model’s contention that heuristic and systematic processing can occur simultaneously to affect judgments when they are congruent [3, 30–31]. This finding is inconsistent, however, with the ELM and other findings demonstrating that heuristic/peripheral information has no effect when systematic/central processing occurs (e.g., [2, 13, 49–50]. It might be the case that this facet of the ELM does not apply to groups comprising a set of individuals with diverse cognitive styles. That is, group processing that requires individuals who rely on both central and peripheral information to agree might need concurrent peripheral and central information to do so successfully.

### Practical implications

Our study also has important implications for the legal system. Of course, in an actual trial, there is no definitive ground truth to classify what is an “accurate” verdict—what a jury decides is considered accurate by the legal system, unless the decision is appealed and vacated. By conducting a tightly controlled experiment, however, we were able to define a “scientifically accurate” verdict: a verdict in line with expert testimony based on a high-quality scientific study. This has relevance for actual trials. That is, the legal system is built on the assumption that verdicts follow the best evidence presented in a case, both case facts and scientific evidence that informs the interpretation of case facts. We demonstrated that mock jurors’ sensitivity to scientific evidence quality can be improved through cross-examination that highlights flaws in low-quality scientific evidence, which is consistent with some prior experiments [51–53], but contradicts others (e.g., [54–55]). We discovered that cross-examination that highlighted flaws in the weak expert’s message was helpful, in part, because it made deliberation more productive. This suggests a potential explanation for why previous studies omitting deliberation found cross-examination ineffective in this regard [54–55]. Importantly, the cross-examination that included central information about the experts sensitized the mock jurors to the quality discrepancy between the two experts, which led them to more scientifically accurate verdicts without making them skeptical of both experts, which has been a concern in cases with dueling experts (e.g.,[56]). Attorneys are often advised to avoid addressing flaws in the expert’s science unless they are as knowledgeable about how the expert’s work hurts their case as the expert is (e.g., [36]). Further, attorneys have difficulty distinguishing between low and high quality science and effectively bringing this out in cross-examination [57]. Given these findings, along with previous studies (that did not include deliberation) that argue cross-examination is not an effective way to sensitize jurors to expert quality, it is important to inform attorneys that their cross-examination efforts can actually be effective.

Further, the coding of all comments about expert witnesses demonstrated that mock jurors spent almost half of the time discussing the expert testimony (41%). Among those comments about experts, mock juries spent the majority of their time focusing on central (rather than peripheral) information about expert testimony (80%) (see [32], for information about what actual jurors think about experts). These data support Diamond and others’ challenges to critics of the jury who argue that jurors are incompetent and irresponsible, lazily relying on heuristics rather than attempting to evaluating experts’ testimony centrally (e.g., [14, 58]. This conclusion might be weakened if our college sample was overall more motivated than a typical community venire to process information, reflected in higher overall NFC scores. Yet this intuition is not well supported, with some studies finding that students’ NFC scores to be higher than community members’ (e.g., [59]), but others finding the opposite [60–61].

Finally, we provided empirical support for the argument that mock jury versus juror decision-making processes might differ qualitatively [45, 62]. Thus, we have provided an important justification for including deliberation in mock jury studies testing behavior that may be modified through group discussion, given that many argue that deliberation does not have an important impact on verdicts and therefore is not worth the massive time and cost involved.

Although some vilify jurors’ reliance on peripheral heuristics to judge experts’ credibility, jury instructions give jurors free rein to rely on credentials in judging the credibility of experts [14]. In fact, our study revealed that peripheral heuristics can even be helpful to groups who are assessing message strength, because heuristics help jurors who accurately assessed the message quality be more persuasive to a more diverse group—while also demonstrating no danger of helping inaccurate jurors convince juries of the less accurate verdict.

Finally, although applications of our study to legal decision making are most obvious, the work is also applicable to groups reaching consensus on many other issues such as public policy, foreign relations, hiring, etc.

### Strengths, caveats, and future directions

We took great care to set up a complex judgment problem that included both individual and group decision making that could be scored for “accuracy,” and in which we could investigate both measured and manipulated likelihood of relying on central and peripheral information. To ensure internal validity, we specifically controlled not only our cross-examination manipulation, but also the NFC composition of the groups and complexity of the two experts’ testimony, and we counterbalanced several factors to rule out alternative explanations (e.g., whether the plaintiff or defense presented the strong research, which actor played which expert). This enabled us to contribute to the basic social psychological literature regarding dual-process models (e.g., ELM, HSM) in individuals versus groups. Although mock jury studies are never completely representative of the real jury deliberation process, we also took great care to design our study in a manner that would make generalizations to the legal arena possible.

It is very difficult to study actual jurors and juries (but see [32]; The Arizona Jury Project, e.g., [63]), especially when investigating issues that require strict experimental control as did our particular research question. To ensure external validity, we used realistic testimony and legal charges, actual state pattern jury instructions that would be used in such a case, and had lawyers review our materials for realism. We also made sure our participants were jury eligible, and even though they were students, recent work finds negligible differences between student and non-student samples in most mock jury studies [64–66]. Nonetheless, replicating the work with older community members would be useful. We also went beyond many other jury simulations by including audio (rather than written) testimony and jury deliberation (rather than relying on individuals’ judgments), which is rare given the effort they require. This advanced methodology enabled us to contribute to the psychology and law literature regarding mock jurors’ ability to evaluate science in the courtroom, as well as potential interventions to improve this ability.

Replication is always important, and there are many interesting variations of the current study that were beyond the scope of this already complex methodology, but which would make excellent future studies. For example, it could be argued that one should assess whether participants’ comments were (a) favorable or unfavorable to the expert, (b) about the strong or weak expert, or (c) expressing agreement or disagreement with other mock jurors—and surely many others. We did not do so because these added layers would make an already very complex analysis even more complicated, and would result in coding categories containing too few comments on which to conduct meaningful analyses—but also because it was not necessary to test the theory we set out to test. The extent to which jurors focus on central versus peripheral information about experts should predict their accuracy regardless of the expert on which they are focusing. For example, if jurors are focusing on central information about the experts, that process should lead them to better assess testimony quality—and that theory should hold regardless of whether they are talking about how strong the strong expert’s study was, about how weak the weak expert’s study was, or incorrectly stating that the strong expert’s study is weak and then being corrected, etc.

It might also be argued that it would be informative to manipulate the NFC composition of the juries to include all high NFC participants versus all low NFC participants. We did not do so for external validity purposes and because this comparison was not necessary to test our theory. Further, we arranged mock juries as we did to test our theory that jurors who were arguing for a more scientifically accurate verdict would be more persuasive to cognitively diverse groups (i.e., half high NFC, half low NFC) when they augmented their central information with peripheral information.

Ours was a first step in demonstrating that scientifically accurate participants are more persuasive to their jury when using peripheral information, which makes the group more accurate. Important next steps could be to investigate *why* peripheral information was more persuasive when made by jurors arguing for the more scientifically accurate verdict, or to compare a cross-examination condition that included only central information against the weak expert to the two cross-examination conditions in the present study. Our study was a single study representing large-scale, in-depth, exploratory research that is rigorous and can serve as a catalyst for future researchers to pursue novel hypotheses (e.g., [67]) inspired by a rare investigation of dual process models (i.e., ELM, HSM) at the group level.

### Conclusion

The current study expanded previous investigations of seminal dual-process models of persuasion by integrating individual- and group-level processes within one study and analyzing the central versus peripheral content of mock deliberation comments. In doing so, we identified a novel psychological phenomenon that might not be predicted or captured by current dual-process theories of individual decision-making. Peripheral heuristics about a persuasion source, which typically detract from *individuals'* discrimination between strong and weak arguments, had an ironically helpful effect on *groups'* assessment of argument strength when used in conjunction with central information. Despite being intentionally unrelated to message quality, peripheral information served an important purpose during intragroup persuasion: It made group members who were arguing for the more scientifically accurate decision more persuasive to their fellow group members, which in turn, made the group more likely to agree on the scientifically accurate decision. Thus, although peripheral information reduces decision quality at the individual level, it can improve the quality of group decisions—as long as it is in the right hands.

## Supporting information

S1 File Supplemental Materials. All experimental materials and measures utilized in the reported study.
(PDF)
