# Supplementary material for: Individual versus group decision making: Jurors’ reliance on central and peripheral information to evaluate expert testimony
Source: PLoS One. 2017 Sep 20;12(9):e0183580. doi: 10.1371/journal.pone.0183580 (PMC5606931; doi:10.1371/journal.pone.0183580)
Supplement: S1 File — All experimental materials and measures utilized in the reported study. (PDF) [file pone.0183580.s001.pdf]

**Supplementary Materials: Detailed Materials**

**Character Sheet**

*Mark Stevens v. Keen Company*

This sheet describes all of the characters you will hear on the trial recording. You will be able to keep this with you throughout the study to help you follow along and keep track of the characters. Please refer to this sheet when answering questions about the characters.

| **PLAINTIFF'S SIDE** | **DEFENSE'S SIDE** |
|---|---|
| ***Mark Stevens***, the plaintiff, is suing Keen Company for negligence. Mark Stevens claims that Keen Company exposed him to PCBs (chemicals) which caused him to develop cancer. | ***Keen Company,*** the defendant, is being sued by Mark Stevens for negligence. Keen Company claims that the PCB exposure did not cause his cancer. |
| ***Dr. Fallon*** is an expert witness who will testify that PCBs cause cancer. | ***Dr. Campbell*** is an expert witness who will testify that PCBs do NOT cause cancer. |

Here's what you will hear on the trial recording:

**1.** The judge will read a summary of the trial.

**2.** The **plaintiff's expert witness**, ***Dr. Fallon***, will be questioned by the plaintiff's lawyer. **Dr. Fallon** will testify that PCBs **DO** cause cancer.

**3.** The **plaintiff's expert witness**, ***Dr. Fallon***, will be cross-examined by the defense's lawyer.

**4.** The **defense's expert witness**, ***Dr. Campbell***, will be questioned by the defense's lawyer. **Dr. Campbell** will testify that PCBs **DO NOT** cause cancer.

**5.** The **defense's expert witness, *Dr. Campbell***, will be cross-examined by the plaintiff's lawyer.

**Judge's Summary**

Before we begin today, let me give you some very brief background information, which has been agreed upon by both the plaintiff and defense. This suit was filed in 2004 against the defendant, Keen Company, by the plaintiff, Mark A. Stevens, who claims that Keen Company's negligence resulted in his illness. Mark Stevens was diagnosed as suffering from colon cancer at the age of 28, ten years after beginning his employment at Keen Company.

The Plaintiff, Mark Stevens, claims that the cancer was caused by workplace exposure to polychlorinated biphenyls ("PCBs") in heat transfer fluids and through soil contamination at the Keen Company plant. PCBs are used in capacitors and transformers at Keen Company. The EPA and the National Institute of Occupational Safety and Health studied the Keen Company factory and found that there were high amounts of PCB contamination in the workplace. Although Keen had stopped using PCBs in their equipment back in 1996, they didn't clean up the contaminated sites until early 1999. Whether he was exposed is not contested – both sides agree that he was exposed to PCBs in the workplace and that he had 50 times the normal amount of PCBs in his blood. What is in question is whether that exposure to PCBs caused Mr. Stevens' cancer.

You will hear only parts of the trial. You will hear testimony from two expert witnesses. One expert for the **Plaintiff**, named ***Dr. Thomas Fallon***, a biochemist at the University of Indiana Medical School, will testify that PCBs *cause* colon cancer. An expert for the **Defense**, Dr. ***William R. Campbell***, a biochemist at University of Michigan, will testify for the defense that PCBs *do NOT* cause cancer.

## Trial Transcripts

**<u>VERSION 1:</u> *Low-quality* plaintiff expert science & *high-quality* defense expert science with *"Peripheral* + *Central"* cross-examination:**

**Expert Witness #1 testimony for the Plaintiff: Dr. Thomas Fallon**
Question from Mark Stevens' (the plaintiff) lawyer: Doctor, would you please state your name for the record.
Expert witness for the plaintiff's answer: My name is Dr. Thomas Fallon.

Q: And what is your profession?
A: I am a Professor and Biochemist at the University of Indiana Medical School, specializing in the effects of carcinogens in animals.

Q: So, you do cancer research?
A: Yes.

Q: Doctor, can you tell us your educational background?
A: I graduated from Johns Hopkins in 1980 with a double major in biology and chemistry. I received my PhD in Biochemistry from University of Oregon in 1984. I am currently a biochemist at the University of Indiana Medical School. I head a laboratory in which I study the effects of PCBs and other carcinogens on animals. I teach graduate courses on the effects of chemicals on living organisms. I have published 42 articles on cancer in peer-reviewed journals, two books, and have made over 100 presentations at national conferences.

Q: (To the judge): At this time, Your Honor, the Plaintiff, Mark Stevens, wishes to qualify Doctor Thomas Fallon as an expert in the field of biochemistry.

Judge: Does the Defendant, Keen Co., have any objections?

Defense Attorney: No, Your Honor.
Judge: You may proceed.

Q: So, Doctor Fallon, you do research on substances that cause cancer?
A: Yes. In my laboratory I study several carcinogens, including PCBs.

Q: So PCBs are carcinogenic.
A: Yes, PCBs cause cancer.

Q: In your opinion, did Mark Stevens contract cancer from his exposure to PCBs?
A: Yes, I believe he did.

Q: Can you explain to us the value of animal studies? Why do you use animals?
A: We use animals to study the effects of dangerous chemicals because we can't use people. Laboratory animals are also easy and inexpensive to grow and maintain, they grow up quickly and have short life-spans, therefore you are able to conduct life span experiments quickly.

Q: So how do you conduct this research?
A: What we usually do is carefully control the amount of a chemical (in this case PCBs) that a group of animals receives and then follow the animals and see whether they develop cancer and other health problems over a period of time.

Q: Could you please describe for us a specific study your lab has conducted and explain the results?
A: In my studies, I expose animals to PCBs and observe the effects on the animals, more specifically whether or not they develop cancer. For example, one experiment involved a group of 15 rats who all received a large dose of Arochlor 1254 (the kind of PCB Mark Stevens was exposed to). We then observed the rats for a year to determine whether or not they developed cancer. We found a high cancer rate in rats exposed to PCBs.

Q: What exactly did you observe?
A: After one year, a high number of the rats who were exposed to PCBs had different types of cancer. These results are currently under review for publication in Neurology & Toxicology.

Q: Doctor Fallon, how many studies like this have you conducted?
A: My lab has published about 42 papers. Out of those, we probably published 27 papers on animal studies, and probably 15 papers were on PCBs in particular.

Q: Doctor Fallon, everything you've told us here today suggests very strongly that colon cancer can result from exposure to PCBs. Is there anything else that you believe would support your statement?
A: Yes. I looked at others who worked at Keen Co. who developed cancer around the same time as Mark Stevens, and I found 47 people. Of those, seven workers were diagnosed with cancer. This is much more than would ever be expected by chance alone.

Q: Doctor, of these seven men at the Keen plant who got cancer, what kinds of cancer did they get?
A: There were two people with colon cancer, one person who had lung cancer, one person with liver cancer, and three other kinds.

Q: Doctor, why would one carcinogen cause so many kinds of tumors?
A: The men might have been exposed in different ways. That is, the PCBs might have come to these men through the air, the water, the soil, or through contact with their skin. So the PCBs might have gotten into the bodies in different ways, and that might have caused the different cancers.

Q: And there is no reason to believe that Mark would have contracted cancer had he not been exposed to PCBs?
A: That is also correct.

Q: Doctor Fallon, can you give us your expert opinion as to the cause of Mark's colon cancer?

A: In my expert opinion, Mark Stevens' colon cancer was caused by the exposure to PCBs at the Keen company plant.

Q: Thank you. That is all, Your Honor.

**Cross-examination of Dr. Fallon:**
The defendant's (Keen Co.) lawyer will now have a chance to cross-examine the plaintiff's (Mark Stevens) expert witness, Dr. Fallon.

Question from the defendant's (Keen Company) lawyer: Doctor Fallon, is it true that you are not a lawyer?
Dr. Fallon's answer: That is correct.

Q: So, you are not a legal expert who can testify as to whether the company is legally responsible for Mark Stevens' illness?
A: No, I cannot speak to that at all. I am not an expert about legal matters.

Q: So, your opinion as to whether Keen Co is responsible for Mark Steven's illness is irrelevant?
A: Well, my opinion about whether PCBs cause cancer is very relevant.

Q: Of course, but when you said during your testimony that it is your expert opinion that PCBs caused Mark Stevens' illness, that was somewhat misleading, wasn't it?
A: How so?

Q: Well, your research is about whether PCBs cause cancer generally, but your research was not specific to Mark Stevens' case, was it?
A: No, it was not.

Q: Can you predict any specific individual's likelihood of developing cancer as a result of PCBs from your study's results?
A: No, I cannot.

Q: So, how much were you paid to be here today?
A: I don't think that is relevant.

Q: You are, in fact, paid to be here today, is this correct?
A: Yes, I am. I am paid to educate people in many different ways, this is no exception. This is my job.

Q: And can you tell us who paid for you to be here today?
A: The plaintiff.

Q: And how often do you get paid to testify?
A: I do not do this for the money if that is what you are inferring. I do not need the money.

Q: Some experts testify for free to avoid that confusion, don't they?

A: Yes.

Q: But not you.
A: No. I do not testify for free, but many expert witnesses accept compensation for their testimony.

Q: How often do you testify, Dr. Fallon?
A: I typically testify a few times a year.

Q: How many times have you testified overall?
A: I have testified in 45 cases.

Q: Do you always testify for the plaintiff?
A: No, I do not…I have testified in cases as an expert for the plaintiff many times, but I have also testified for the defense in cases where I do not believe the defendant's illness was caused by PCBs.

Q: How many times has this happened – where you testify for the defense?
A: I have testified for the defense 7 times.

Q: So, you testify for the plaintiff side 84% of the time.
A: Yes, thus far.

Q: So, would you call yourself more of a plaintiff witness?
A: No, I would not. I testify for the side that I believe in.

Q: So, 84% of cases involving PCBs just happen to be strong plaintiff cases?
A: I believe it to be a dangerous substance. I am surprised there aren't more.

Q: Doctor, let me address another issue. Could you please explain what a control group is in an experiment?
A: A control group is a group that does not receive the variable that you are studying. The control group is identical in every way to the experimental group except it lacks the thing you are studying. That way when you compare the two groups you can confidently say that the difference is due to the only thing that was different, the variable.

Q: In your study, what would your control group be?
A: The control group would be a group of rats that did not receive the PCBs.

Q: Did you use a control group in your study?
A: No.

Q: Then couldn't it be that you had an unhealthy group of rats that got the cancer for some other reason? Maybe those rats experienced something else in the environment that could be causing cancer?
A: Yes, that is a possibility…though very unlikely.

Q: But without a control group, you can't say for sure can you?
A: No.

Q: And has this study been through peer review and published?
A: It is in the process. It is a very important study and one of the top journals in the field is currently reviewing it.

Q: Can you explain what peer review means?
A: No article can be published until other scientists in the field have read it and had a chance to ask questions, make you revise it, or to reject it.

Q: Why would an article not pass peer review?
A: If the methods used in the study are judged to be flawed by one's peers.

Q: But, there has been no opportunity for anyone to read your article and voice concerns about it?
A: No, that process has not been completed yet.

Q: Ok, let's go to another topic. Can you please remind us how your rats were exposed to the PCBs in your study?
A: Yes, the rats are given a large dose of PCBs and then observed and tested for cancer; the standard procedure in the field.

Q: The Defense witness will testify that there is NOT a link between PCBs and cancer. However, he exposed his rats differently. He exposed his rats with smaller doses over a longer period of time? Why didn't you do that?
A: Well, exposing rats that way is not the standard way of doing this in the field. This is how I have always exposed my rats.

Q: Do you feel that your way more similar to the way Mark Stevens was exposed to PCBs?
A: No, he was exposed in small amounts over a longer period of time.

Q: So, had you exposed the rats the way Mark Stevens was exposed, you could have also failed to find a link between PCBs and cancer?
A: I certainly have no idea what the results would be.

Q: No further questions, Your Honor.

**Expert Witness #2 for the Defense: Dr. William Campbell**
Judge: We are now ready to proceed with the Defense's witness. Are you ready with your witness?
Keen Co.'s (the defendant) lawyer: Yes, Your Honor.

Judge: You may proceed.

Question from Keen Co.'s lawyer: Doctor, would you please state your name and address for the record?
The expert witness for the defense's answer: My name is Dr. William Campbell.

Q: Doctor Campbell, what is your current occupation?
A: I am a professor of Biochemistry at the University of Michigan.

Q: Thank you. And were did you receive your training in this science?
A: I received my Bachelor's degree from Tufts University in 1977 with a concentration in mathematics and biology. I went on to complete my PhD studies in Biochemistry in 1982 at University of Iowa. I am currently a Professor of biochemistry at the University of Michigan. I teach a seminar in Biochemistry to graduate students in public health, and an "Environment and Disease" class to the medical students. I have published over 30 peer reviewed articles in such journals, a book and a number of invited chapters for edited books. I have also presented my work at national conferences on well over 100 occasions in the last 15 years.

Q (To the judge): At this time, Your Honor, the defendant, Keen Co. wishes to qualify Doctor William Campbell as an expert in the field of biochemistry.

Judge: Does the plaintiff, Mark Stevens have any objections?
Howard (plaintiff, Mark Stevens): No, Your Honor.
Judge: Mr. Moore, you may proceed.

Q: Doctor, you have conducted many studies on the effects of PCBs on populations. What have these studies indicated with respect to the association between PCBs and colon cancer?
A: I have found that exposure to PCBs does not cause any increase in the rates of colon cancer.

Q: So animals who were exposed to PCBs were no more likely to contract colon cancer than anybody else in your studies?
A: That's right. My research has indicated that animals in contact with PCBs did not show a high incidence of cancer.

Q: But what does that mean for the plaintiff, Mark Stevens?
A: What that means is that it is unlikely his colon cancer came from PCBs.

Q: So in your expert opinion, PCBs did not cause Mark Stevens' colon cancer?
A: That's right. I believe that PCBs did not cause Mark Stevens' colon cancer.

Q: Is it correct to say that PCBs have not been shown to cause cancer, but they have not been ruled out as a cause of cancer?
A: I think it's stronger than that. There really is very little evidence that PCBs are related to the cancer problem. Although PCBs are poisonous and will kill a person immediately if digested in a large quantity, there is no evidence that PCBs cause cancer in humans.

Q: I see. Doctor, can you tell the jury how you conduct your research and why you are so confident about this lack of connection between PCBs and cancer?

A: I conduct experimental studies on animals to assess whether PCBs cause cancer.

Q: Could you please describe for us a specific study your lab has conducted and explain the results?
A: In one study, we randomly assigned a group of rats to receive a set dose of PCB, Arochlor 1254 was the exact type of PCB, while another group of rats did not receive the PCBs. We exposed the rats to PCBs for one month. Then we stopped and waited for a year. After a year, we compared the rates of cancer for the rats that ate PCBs to the rate of cancer for the rats and those in the control group that did not ingest PCBs. We found that the rats exposed to PCBs did not have a higher rate of cancer than those in the control group. This cancer rate was not significantly different from the group of rats that were not exposed to PCBs. These results were published in Neurology & Toxicology.

Q: Can you give the jury further details about how you go about conducting these experiments?
A: Of course. For the first phase of the study we exposed the rats to a small amount of PCBs every day for a prolonged period of time, similar to the exposure Mark Stevens would have undergone at Keen Company. The exposure was small, but steady, we exposed the rats every day for a month. The control group was exposed to a completely harmless dose of saline every day for a month. After this period was over, we observed the rats for a year, after which we tested for cancer and recorded the rate of occurrence.

Q: Are you aware that the plaintiff's witness is testifying that his study found contrary results? How do you explain the fact that his animal study found that PCBs caused cancer while your study did not?
A: Animal studies are only useful when one is only talking about the same kind of cancer in humans. For example, it would be reasonable to use a study's findings that PCBs caused liver cancer in mice to say that humans may also be afflicted with liver cancer. But we can only do this when we are talking about the same type of cancer. You can't compare across different cancers.

Q: So, if a study found that PCBs caused liver cancer in rats, it would be improper in your opinion, to say that PCBs caused colon cancer in humans?
A: Exactly. Just because PCBs might cause liver cancer in rats, you can't say whether or not PCBs might cause colon cancer in humans. They're two different organs and species, it's just not the same.

Q: Then, in your opinion, would you say that PCBs were not the cause of the plaintiff, Mark Stevens' illness?
A: Yes, it is very unlikely that PCBs caused the plaintiff, Mark Stevens' colon cancer.

Q: Thank you very much, Doctor.

**Cross-examination of Dr. Campbell:**
The plaintiff's (Mark Stevens) lawyer will now have a chance to cross-examine the defendant's (Keen Co.) expert witness, Dr. Campbell.

Judge: You may cross-examine the witness.

Mark Stevens' lawyer: Thank you, Your Honor.

Question from Mark Stevens' lawyer: Dr. Campbell, do you always testify for the defense?
Dr. Campbell's answer: No, I do not… I have testified for the plaintiff side before.

Q: Why would you do that, if your studies show that PCBs do not cause cancer?
A: Well, occasionally there are cases where it is obvious that the person's illness was caused by PCBs.

Q: Would you say it is important for an expert to not always side with the same side in a court case?
A: Yes, I would agree with that.

Q: Why do you feel that is important?
A: Well, one does not want to become a hired gun for one side…an expert should stay balanced.

Q: And do you consider yourself balanced?
A: Well, I'd like to find more cases for the plaintiff to testify for, but I find that I tend to mostly find cases in which I believe in the defense's side.

Q: How many times have you testified for the plaintiff?
A: 10 times.

Q: Out of how many?
A: Roughly 90 cases.

Q: So, you have testified for the defense 90% of the time?
A: That is correct. There aren't many cases in which I believe PCBs caused the cancer.

Q: Doctor Campbell, isn't it true that your research has been criticized by your peers?
A: No, that is not true. I am very well respected in my field.

Q: Are you saying that there is no dispute in your field about the accuracy of your study that found that PCBs cause cancer?
A: Well, some scientists do disagree with me, but that is not based on the quality of my work.

Q: What would it be based on then?
A: Different researchers conduct their research differently and as such, they find different results. All researchers find themselves needing to defend their methods and their conclusions at some point or another. I have not had to do so any more than any other researcher in the field.

Q: How can you be sure that your way is correct and that those who find different results are wrong?

A: I stand by my methods and my research… as do the many journals and publishers that have published my work. Articles must go through a rigorous peer review process before they are published. If my methods were flawed, I would not have been published.

Q: Would you say that everything that is published should be trusted?
A: No, there are some lower level journals that have lower standards.

Q: Are all of your studies published in the top journal in the field?
A: No, of course not all of them, but some of them are. I can assure you, all the journals I publish in are respected journals that can be trusted.

Q: Are you paid to testify today doctor?
A: Yes, of course. All experts are offered compensation for their testimony. We are providing a valuable service to the court.

Q:And is it the court that pays you?
A: No, it is not.

Q: Who pays you then?
A: The defense pays me.

Q: No further questions your honor.

**VERSION 2: Low-Quality plaintiff expert & high-quality defense expert with "*Peripheral only*" cross-examination:**

**Expert Witness #1 testimony for the Plaintiff: Dr. Thomas Fallon**
Question from Mark Stevens' (the plaintiff) lawyer: Doctor, would you please state your name for the record.
Expert witness for the plaintiff's answer: My name is Dr. Thomas Fallon.

Q: And what is your profession?
A: I am a Professor and Biochemist at the University of Indiana Medical School, specializing in the effects of carcinogens in animals.

Q: So, you do cancer research?
A: Yes.

Q: Doctor, can you tell us your educational background?
A: I graduated from Johns Hopkins in 1980 with a double major in biology and chemistry. I received my PhD in Biochemistry from University of Oregon in 1984. I am currently a biochemist at the University of Indiana Medical School. I head a laboratory in which I study the effects of PCBs and other carcinogens on animals. I teach graduate courses on the effects of chemicals on living organisms. I have published 42 articles on cancer in peer-reviewed journals, two books, and have made over 100 presentations at national conferences.

Q (To the judge): At this time, Your Honor, the Plaintiff, Mark Stevens, wishes to qualify Doctor Thomas Fallon as an expert in the field of biochemistry.

Judge: Does the Defendant, Keen Co., have any objections?

Defense Attorney: No, Your Honor.
Judge: You may proceed.

Q: So, Doctor Fallon, you do research on substances that cause cancer?
A: Yes. In my laboratory I study several carcinogens, including PCBs.

Q: So PCBs are carcinogenic.
A: Yes, PCBs cause cancer.

Q: In your opinion, did Mark Stevens contract cancer from his exposure to PCBs?
A: Yes, I believe he did.

Q: Can you explain to us the value of animal studies? Why do you use animals?
A: We use animals to study the effects of dangerous chemicals because we can't use people. Laboratory animals are also easy and inexpensive to grow and maintain, they grow up quickly and have short life-spans, therefore you are able to conduct life span experiments quickly.

Q: So how do you conduct this research?

A: What we usually do is carefully control the amount of a chemical (in this case PCBs) that a group of animals receives and then follow the animals and see whether they develop cancer and other health problems over a period of time.

Q: Could you please describe for us a specific study your lab has conducted and explain the results?
A: In my studies, I expose animals to PCBs and observe the effects on the animals, more specifically whether or not they develop cancer. For example, one experiment involved a group of 15 rats who all received a large dose of Arochlor 1254 (the kind of PCB Mark Stevens was exposed to). We then observed the rats for a year to determine whether or not they developed cancer. We found a high cancer rate in rats exposed to PCBs.

Q: What exactly did you observe?
A: After one year, a high number of the rats who were exposed to PCBs had different types of cancer. These results are currently under review for publication in *Neurology & Toxicology*.

Q: Doctor Fallon, how many studies like this have you conducted?
A: My lab has published about 42 papers. Out of those, we probably published 27 papers on animal studies, and probably 15 papers were on PCBs in particular.

Q: Doctor Fallon, everything you've told us here today suggests very strongly that colon cancer can result from exposure to PCBs. Is there anything else that you believe would support your statement?
A: Yes. I looked at others who worked at Keen Co. who developed cancer around the same time as Mark Stevens, and I found 47 people. Of those, seven workers were diagnosed with cancer. This is much more than would ever be expected by chance alone.

Q: Doctor, of these seven men at the Keen plant who got cancer, what kinds of cancer did they get?
A: There were two people with colon cancer, one person who had lung cancer, one person with liver cancer, and three other kinds.

Q: Doctor, why would one carcinogen cause so many kinds of tumors?
A: The men might have been exposed in different ways. That is, the PCBs might have come to these men through the air, the water, the soil, or through contact with their skin. So the PCBs might have gotten into the bodies in different ways, and that might have caused the different cancers.

Q: And there is no reason to believe that Mark would have contracted cancer had he not been exposed to PCBs?
A: That is also correct.

Q: Doctor Fallon, can you give us your expert opinion as to the cause of Mark's colon cancer?
A: In my expert opinion, Mark Stevens' colon cancer was caused by the exposure to PCBs at the Keen company plant.

Q: Thank you. That is all, Your Honor.

**Cross-examination of Dr. Fallon:**
Question from the defendant's (Keen Company) lawyer: Doctor Fallon, is it true that you are not a lawyer?
Dr. Fallon's answer: That is correct.

Q: So, you are not a legal expert who can testify as to whether the company is legally responsible for Mark Stevens' illness?
A: No, I cannot speak to that at all. I am not an expert about legal matters.

Q: So, your opinion as to whether Keen Co is responsible for Mark Steven's illness is irrelevant?
A: Well, my opinion about whether PCBs cause cancer is very relevant.

Q: Of course, but when you said during your testimony that it is your expert opinion that PCBs caused Mark Stevens' illness, that was somewhat misleading, wasn't it?
A: How so?

Q: Well, your research is about whether PCBs cause cancer generally, but your research was not specific to Mark Stevens' case, was it?
A: No, it was not.

Q: Can you predict any specific individual's likelihood of developing cancer as a result of PCBs from your study's results?
A: No, I cannot.

Q: So, how much were you paid to be here today?
A: I don't think that is relevant.

Q: You are, in fact, paid to be here today, is this correct?
A: Yes, I am. I am paid to educate people in many different ways, this is no exception. This is my job.

Q: And can you tell us who paid for you to be here today?
A: The plaintiff.

Q: And how often do you get paid to testify?
A: I do not do this for the money if that is what you are inferring. I do not need the money.

Q: Some experts testify for free to avoid that confusion, don't they?
A: Yes.

Q: But not you.
A: No. I do not testify for free, but many expert witnesses accept compensation for their testimony.

Q: How often do you testify, Dr. Fallon?
A: I typically testify a few times a year.

Q: How many times have you testified overall?
A: I have testified in 45 cases.

Q: Do you always testify for the plaintiff?
A: No, I do not…I have testified in cases as an expert for the plaintiff many times, but I have also testified for the defense in cases where I do not believe the defendant's illness was caused by PCBs.

Q: How many times has this happened – where you testify for the defense?
A: I have testified for the defense 7 times.

Q: So, you testify for the plaintiff side 84% of the time.
A: Yes, thus far.

Q: So, would you call yourself more of a plaintiff witness?
A: No, I would not. I testify for the side that I believe in.

Q: So, 84% of cases involving PCBs just happen to be strong plaintiff cases?
A: I believe it to be a dangerous substance. I am surprised there aren't more.

Q: No further questions, Your Honor.

**Expert Witness #2 for the Defense: Dr. William Campbell**
Judge: We are now ready to proceed with the Defense's witness. Are you ready with your witness?
Keen Co.'s (the defendant) lawyer: Yes, Your Honor.
Judge: You may proceed.

Question from Keen Co.'s lawyer: Doctor, would you please state your name and address for the record?
The expert witness for the defense's answer: My name is Dr. William Campbell.

Q: Doctor Campbell, what is your current occupation?
A: I am a professor of Biochemistry at the University of Michigan.

Q: Thank you. And were did you receive your training in this science?
A: I received my Bachelor's degree from Tufts University in 1977 with a concentration in mathematics and biology. I went on to complete my PhD studies in Biochemistry in 1982 at University of Iowa. I am currently a Professor of biochemistry at the University of Michigan.
I teach a seminar in Biochemistry to graduate students in public health, and an "Environment and Disease" class to the medical students. I have published over 30 peer reviewed articles in such journals, a book and a number of invited chapters for edited books. I have also presented my work at national conferences on well over 100 occasions in the last 15 years.

Q (To the judge): At this time, Your Honor, the defendant, Keen Co. wishes to qualify Doctor William Campbell as an expert in the field of biochemistry.

Judge: Does the plaintiff, Mark Stevens have any objections?
Howard (plaintiff, Mark Stevens): No, Your Honor.
Judge: Mr. Moore, you may proceed.

Q: Doctor, you have conducted many studies on the effects of PCBs on populations. What have these studies indicated with respect to the association between PCBs and colon cancer?
A: I have found that exposure to PCBs does not cause any increase in the rates of colon cancer.

Q: So people who were exposed to PCBs were no more likely to contract colon cancer than anybody else in your studies?
A: That's right. My research has indicated that animals in contact with PCBs did not show a high incidence of cancer.

Q: But what does that mean for the plaintiff, Mark Stevens?
A: What that means is that it is unlikely his colon cancer came from PCBs.

Q: So in your expert opinion, PCBs did not cause Mark Stevens' colon cancer?
A: That's right. I believe that PCBs did *not* cause Mark Stevens' colon cancer.

Q: Is it correct to say that PCBs have not been shown to cause cancer, but they have not been ruled out as a cause of cancer?
A: I think it's stronger than that. There really is very little evidence that PCBs are related to the cancer problem. Although PCBs are poisonous and will kill a person immediately if digested in a large quantity, there is no evidence that PCBs cause cancer in humans.

Q: I see. Doctor, can you tell the jury how you conduct your research and why you are so confident about this lack of connection between PCBs and cancer?
A: I conduct experimental studies on animals to assess whether PCBs cause cancer.

Q: Could you please describe for us a specific study your lab has conducted and explain the results?
A: In one study, we randomly assigned a group of rats to receive a set dose of PCB, Arochlor 1254 was the exact type of PCB, while another group of rats did not receive the PCBs. We exposed the rats to PCBs for one month. Then we stopped and waited for a year. After a year, we compared the rates of cancer for the rats that ate PCBs to the rate of cancer for the rats and those in the control group that did not ingest PCBs. We found that the rats exposed to PCBs did not have a higher rate of cancer than those in the control group. This cancer rate was not significantly different from the group of rats that were not exposed to PCBs. These results were published in *Neurology & Toxicology*.

Q: Can you give the jury further details about how you go about conducting these experiments?

A: Of course. For the first phase of the study we exposed the rats to a small amount of PCBs every day for a prolonged period of time, similar to the exposure Mark Stevens would have undergone at Keen Company. The exposure was small, but steady, we exposed the rats every day for a month. The control group was exposed to a completely harmless dose of saline every day for a month. After this period was over, we observed the rats for a year, after which we tested for cancer and recorded the rate of occurrence.

Q: Are you aware that the plaintiff's witness is testifying that his study found contrary results? How do you explain the fact that his animal study found that PCBs caused cancer while your study did not?
A: Animal studies are only useful when one is only talking about the same kind of cancer in humans. For example, it would be reasonable to use a study's findings that PCBs caused liver cancer in mice to say that humans may also be afflicted with liver cancer. But we can only do this when we are talking about the same type of cancer. You can't compare across different cancers.

Q: So, if a study found that PCBs caused liver cancer in rats, it would be improper in your opinion, to say that PCBs caused colon cancer in humans?
A: Exactly. Just because PCBs might cause liver cancer in rats, you can't say whether or not PCBs might cause colon cancer in humans. They're two different organs and species, it's just not the same.

Q: Then, in your opinion, would you say that PCBs were not the cause of the plaintiff, Mark Stevens' illness?
A: Yes, it is very unlikely that PCBs caused the plaintiff, Mark Stevens' colon cancer.

Q: Thank you very much, Doctor.

**Cross-examination of Dr. Campbell:**
Judge: You may cross-examine the witness.

Mark Stevens' lawyer: Thank you, Your Honor.

Question from Mark Stevens' lawyer: Dr. Campbell, do you always testify for the defense?
Dr. Campbell's answer: No, I do not… I have testified for the plaintiff side before.

Q: Why would you do that, if your studies show that PCBs do not cause cancer?
A: Well, occasionally there are cases where it is obvious that the person's illness was caused by PCBs.

Q: Would you say it is important for an expert to not always side with the same side in a court case?
A: Yes, I would agree with that.

Q: Why do you feel that is important?
A: Well, one does not want to become a hired gun for one side…an expert should stay balanced.

Q: And do you consider yourself balanced?
A: Well, I'd like to find more cases for the plaintiff to testify for, but I find that I tend to mostly find cases in which I believe in the defense's side.

Q: How many times have you testified for the plaintiff?
A: 10 times.

Q: Out of how many?
A: Roughly 90 cases.

Q: So, you have testified for the defense 90% of the time?
A: That is correct. There aren't many cases in which I believe PCBs caused the cancer.

Q: Doctor Campbell, isn't it true that your research has been criticized by your peers?
A: No, that is not true. I am very well respected in my field.

Q: Are you saying that there is no dispute in your field about the accuracy of your study that found that PCBs cause cancer?
A: Well, some scientists do disagree with me, but that is not based on the quality of my work.

Q: What would it be based on then?
A: Different researchers conduct their research differently and as such, they find different results. All researchers find themselves needing to defend their methods and their conclusions at some point or another. I have not had to do so any more than any other researcher in the field.

Q: How can you be sure that your way is correct and that those who find different results are wrong?
A: I stand by my methods and my research… as do the many journals and publishers that have published my work. Articles must go through a rigorous peer review process before they are published. If my methods were flawed, I would not have been published.

Q: Would you say that everything that is published should be trusted?
A: No, there are some lower level journals that have lower standards.

Q: Are all of your studies published in the top journal in the field?
A: No, of course not all of them, but some of them are. I can assure you, all the journals I publish in are respected journals that can be trusted.

Q: Are you paid to testify today doctor?
A: Yes, of course. All experts are offered compensation for their testimony. We are providing a valuable service to the court.

Q:And is it the court that pays you?
A: No, it is not.

Q: Who pays you then?
A: The defense pays me.

Q: No further questions your honor.

**VERSION 3:** ***High-quality*** **plaintiff expert science &** ***low-quality*** **defense expert science with** ***"Peripheral + Central"*** **cross-examination:**

**Expert Witness #1 testimony for the Plaintiff: Dr. Thomas Fallon**
*[Question from Mark Stevens' (the plaintiff) lawyer*]: Doctor, would you please state your name for the record.
Expert witness for the plaintiff's answer: My name is Dr. Thomas Fallon.

Q: And what is your profession?
A: I am a Professor and Biochemist at the University of Indiana Medical School, specializing in the effects of carcinogens in animals.

Q: So, you do cancer research?
A: Yes.

Q: Doctor, can you tell us your educational background?
A: I graduated from Johns Hopkins in 1980 with a double major in biology and chemistry. I received my PhD in Biochemistry from University of Oregon in 1984. I am currently a biochemist at the University of Indiana Medical School. I head a laboratory in which I study the effects of PCBs and other carcinogens on animals. I teach graduate courses on the effects of chemicals on living organisms. I have published 42 articles on cancer in peer-reviewed journals, two books, and have made over 100 presentations at national conferences.

Q (To the judge): At this time, Your Honor, the Plaintiff, Mark Stevens, wishes to qualify Doctor Thomas Fallon as an expert in the field of biochemistry.

Judge: Does the Defendant, Keen Co., have any objections?

Defense Attorney: No, Your Honor.
Judge: You may proceed.

Q: So, Doctor Fallon, you do research on substances that cause cancer?
A: Yes. In my laboratory I study several carcinogens, including PCBs.

Q: So PCBs are carcinogenic.
A: Yes, PCBs cause cancer.

Q: In your opinion, did Mark Stevens contract cancer from his exposure to PCBs?
A: Yes, I believe he did.

Q: Can you explain to us the value of animal studies? Why do you use animals?
A: We use animals to study the effects of dangerous chemicals because we can't use people. Laboratory animals are also easy and inexpensive to grow and maintain, they grow up quickly and have short life-spans, therefore you are able to conduct life span experiments quickly.

Q: I see. Dr., can you tell the jury how you conduct your research and why you are so confident about this connection between PCBs and cancer?
A: I conduct experimental studies on animals to assess whether PCBs cause cancer.

Q: Could you please describe for us a specific study your lab has conducted and explain the results?
A: In one study, we randomly assigned a group of rats to receive a set dose of PCB, Arochlor 1254 was the exact type of PCB, while another group of rats did not receive the PCBs. We exposed the rats to PCBs for one month. Then we stopped and waited for a year. After a year, we compared the rates of cancer for the rats that ate PCBs and those in the control group that did not. We found that the rats exposed to PCBs had a significantly higher rate of liver cancer than those in the control group.

Q: Can you give the jury further details about how you go about conducting these experiments?
A: Of course. For the first phase of the study we exposed the rats to a small amount of PCBs every day for a prolonged period of time, similar to the exposure Mark Stevens would have undergone at Keen Company. The exposure was small, but steady, we exposed the rats every day for a month. The control group was exposed to a completely harmless dose of saline every day for a month. After this period was over, we observed the rats for a year, after which we tested for cancer and recorded the rate of occurrence.

Q: What exactly did you observe?
A: After one year, the rats who were exposed to PCBs had a significantly higher rate of liver cancer than the rats who were not exposed to PCBs. These results were published in *Neurology & Toxicology*.

Q: Doctor Fallon, how many studies like this have you conducted?
A: My lab has published about 42 papers. Out of those, we probably published 27 papers on animal studies, and probably 15 papers were on PCBs in particular.

Q: Doctor Fallon, everything you've told us here today suggests very strongly that colon cancer can result from exposure to PCBs. Is there anything else that you believe would support your statement?
A: Yes. I looked at others who worked at Keen Co. who developed cancer around the same time as Mark Stevens, and I found 47 people. Of those, seven workers were diagnosed with cancer. This is much more than would ever be expected by chance alone.

Q: Doctor, of these seven men at the Keen plant who got cancer, what kinds of cancer did they get?
A: There were two people with colon cancer, one person who had lung cancer, one person with liver cancer, and three other kinds.

Q: Doctor, why would one carcinogen cause so many kinds of tumors?
A: The men might have been exposed in different ways. That is, the PCBs might have come to these men through the air, the water, the soil, or through contact with their skin. So the PCBs

might have gotten into the bodies in different ways, and that might have caused the different cancers.

Q: And there is no reason to believe that Mark would have contracted cancer had he not been exposed to PCBs?
A: That is also correct.

Q: Doctor Fallon, can you give us your expert opinion as to the cause of Mark's colon cancer?
A: In my expert opinion, Mark Stevens' colon cancer was caused by the exposure to PCBs at the Keen company plant.

Q: Thank you. That is all, Your Honor.

**Cross-examination of Dr. Fallon:**
Question from the defendant's (Keen Company) lawyer: Doctor Fallon, is it true that you are not a lawyer?
Dr. Fallon's answer: That is correct.

Q: So, you are not a legal expert who can testify as to whether the company is legally responsible for Mark Stevens' illness?
A: No, I cannot speak to that at all. I am not an expert about legal matters.

Q: So, your opinion as to whether Keen Co is responsible for Mark Steven's illness is irrelevant?
A: Well, my opinion about whether PCBs cause cancer is very relevant.

Q: Of course, but when you said during your testimony that it is your expert opinion that PCBs caused Mark Stevens' illness, that was somewhat misleading, wasn't it?
A: How so?

Q: Well, your research is about whether PCBs cause cancer generally, but your research was not specific to Mark Stevens' case, was it?
A: No, it was not.

Q: Can you predict any specific individual's likelihood of developing cancer as a result of PCBs from your study's results?
A: No, I cannot.

Q: So, how much were you paid to be here today?
A: I don't think that is relevant.

Q: You are, in fact, paid to be here today, is this correct?
A: Yes, I am. I am paid to educate people in many different ways, this is no exception. This is my job.

Q: And can you tell us who paid for you to be here today?
A: The plaintiff.

Q: And how often do you get paid to testify?
A: I do not do this for the money if that is what you are inferring. I do not need the money.

Q: Some experts testify for free to avoid that confusion, don't they?
A: Yes.

Q: But not you.
A: No. I do not testify for free, but many expert witnesses accept compensation for their testimony.

Q: How often do you testify, Dr. Fallon?
A: I typically testify a few times a year.

Q: How many times have you testified overall?
A: I have testified in 45 cases.

Q: Do you always testify for the plaintiff?
A: No, I do not…I have testified in cases as an expert for the plaintiff many times, but I have also testified for the defense in cases where I do not believe the defendant's illness was caused by PCBs.

Q: How many times has this happened – where you testify for the defense?
A: I have testified for the defense 7 times.

Q: So, you testify for the plaintiff side 84% of the time.
A: Yes, thus far.

Q: So, would you call yourself more of a plaintiff witness?
A: No, I would not. I testify for the side that I believe in.

Q: So, 84% of cases involving PCBs just happen to be strong plaintiff cases?
A: I believe it to be a dangerous substance. I am surprised there aren't more.

**Expert Witness #2 for the Defense: Dr. William Campbell**
Judge: We are now ready to proceed with the Defense's witness. Are you ready with your witness?
Keen Co.'s (the defendant) lawyer: Yes, Your Honor.

Judge: You may proceed.

Question from Keen Co.'s lawyer: Doctor, would you please state your name and address for the record?
The expert witness for the defense's answer: My name is Dr. William Campbell.

Q: Doctor Campbell, what is your current occupation?

A: I am a professor of Biochemistry at the University of Michigan.

Q: Thank you. And were did you receive your training in this science?
A: I received my Bachelor's degree from Tufts University in 1977 with a concentration in mathematics and biology. I went on to complete my PhD studies in Biochemistry in 1982 at University of Iowa. I am currently a Professor of biochemistry at the University of Michigan. I teach a seminar in Biochemistry to graduate students in public health, and an "Environment and Disease" class to the medical students. I have published over 30 peer reviewed articles in such journals, a book and a number of invited chapters for edited books. I have also presented my work at national conferences on well over 100 occasions in the last 15 years.

Q (To the judge): At this time, Your Honor, the defendant, Keen Co. wishes to qualify Doctor William Campbell as an expert in the field of biochemistry.

Judge: Does the plaintiff, Mark Stevens have any objections?
Howard (plaintiff, Mark Stevens): No, Your Honor.
Judge: Mr. Moore, you may proceed.

Q: Doctor, you have conducted many studies on the effects of PCBs on populations. What have these studies indicated with respect to the association between PCBs and colon cancer?
A: I have found that exposure to PCBs does not cause any increase in the rates of colon cancer.

Q: So people who were exposed to PCBs were no more likely to contract colon cancer than anybody else in your studies?
A: That's right. My research has indicated that animals in contact with PCBs did not show a high incidence of cancer.

Q: But what does that mean for the plaintiff, Mark Stevens?
A: What that means is that it is unlikely his colon cancer came from PCBs.

Q: So in your expert opinion, PCBs did not cause Mark Stevens' colon cancer?
A: That's right. I believe that PCBs did *not* cause Mark Stevens' colon cancer.

Q: Is it correct to say that PCBs have not been shown to cause cancer, but they have not been ruled out as a cause of cancer?
A: I think it's stronger than that. There really is very little evidence that PCBs are related to the cancer problem. Although PCBs are poisonous and will kill a person immediately if digested in a large quantity, there is no evidence that PCBs cause cancer in humans.

Q: Could you please describe for us a specific study your lab has conducted and explain the results?
A: My studies expose animals to PCBs and observe the effects on the animals, more specifically whether or not they develop cancer. For example, one experiment involved a group of 15 rats who all received a large dose of Arochlor 1254 (the kind of PCB Mark Stevens was exposed to). We then observed the rats for a year to determine whether or not they developed cancer. We found a high cancer rate in rats exposed to PCBs.

Q: What were your results?
A: We did not find a high incidence of cancer in the rats. These results are currently under review for publication in *Neurology & Toxicology*.

Q: Are you aware that the plaintiff's witness is testifying that his study found contrary results? How do you explain the fact that his animal study found that PCBs caused cancer while your study did not?
A: Animal studies are only useful when one is only talking about the same kind of cancer in humans. For example, it would be reasonable to use a study's findings that PCBs caused liver cancer in mice to say that humans may also be afflicted with liver cancer. But we can only do this when we are talking about the same type of cancer. You can't compare across different cancers.

Q: So, if a study found that PCBs caused liver cancer in rats, it would be improper in your opinion, to say that PCBs caused colon cancer in humans?
A: Exactly. Just because PCBs might cause liver cancer in rats, you can't say whether or not PCBs might cause colon cancer in humans. They're two different organs and species, it's just not the same.

Q: Then, in your opinion, would you say that PCBs were not the cause of the plaintiff, Mark Stevens' illness?
A: Yes, it is very unlikely that PCBs caused the plaintiff, Mark Stevens' colon cancer.

Q: Thank you very much, Doctor.

**Cross-examination of Dr. Campbell:**
Judge: You may cross-examine the witness.

Mark Stevens' lawyer: Thank you, Your Honor.

Question from Mark Stevens' lawyer: Dr. Campbell, do you always testify for the defense?
Dr. Campbell's answer: No, I do not… I have testified for the plaintiff side before.

Q: Why would you do that, if your studies show that PCBs do not cause cancer?
A: Well, occasionally there are cases where it is obvious that the person's illness was caused by PCBs.

Q: Would you say it is important for an expert to not always side with the same side in a court case?
A: Yes, I would agree with that.

Q: Why do you feel that is important?
A: Well, one does not want to become a hired gun for one side…an expert should stay balanced.

Q: And do you consider yourself balanced?

A: Well, I'd like to find more cases for the plaintiff to testify for, but I find that I tend to mostly find cases in which I believe in the defense's side.

Q: How many times have you testified for the plaintiff?
A: 10 times.

Q: Out of how many?
A: Roughly 90 cases.

Q: So, you have testified for the defense 90% of the time?
A: That is correct. There aren't many cases in which I believe PCBs caused the cancer.

Q: Doctor Campbell, isn't it true that your research has been criticized by your peers?
A: No, that is not true. I am very well respected in my field.

Q: Are you saying that there is no dispute in your field about the accuracy of your study that found that PCBs cause cancer?
A: Well, some scientists do disagree with me, but that is not based on the quality of my work.

Q: What would it be based on then?
A: Different researchers conduct their research differently and as such, they find different results. All researchers find themselves needing to defend their methods and their conclusions at some point or another. I have not had to do so any more than any other researcher in the field.

Q: How can you be sure that your way is correct and that those who find different results are wrong?
A: I stand by my methods and my research… as do the many journals and publishers that have published my work. Articles must go through a rigorous peer review process before they are published. If my methods were flawed, I would not have been published.

Q: Would you say that everything that is published should be trusted?
A: No, there are some lower level journals that have lower standards.

Q: Are all of your studies published in the top journal in the field?
A: No, of course not all of them, but some of them are. I can assure you, all the journals I publish in are respected journals that can be trusted.

Q: Are you paid to testify today doctor?
A: Yes, of course. All experts are offered compensation for their testimony. We are providing a valuable service to the court.

Q:And is it the court that pays you?
A: No, it is not.

Q: Who pays you then?
A: The defense pays me.

Q: Doctor, let me address another issue. Could you please explain what a control group is in an experiment?
A: A control group is a group that does not receive the variable that you are studying. The control group is identical in every way to the experimental group except it lacks the thing you are studying. That way when you compare the two groups you can confidently say that the difference is due to the only thing that was different, the variable.

Q: In your study, what would your control group be?
A: The control group would be a group of rats that did not receive the PCBs.

Q: Did your studies use control groups?
A: No.

Q: Couldn't it be that you had an uncharacteristically healthy group of rats that resulted in a lower rate of cancer? Or that there was something else in the environment that all the rats experienced that could be preventing the cancer?
A: Yes, that is a possibility…though very unlikely.

Q: And has this study been through peer review and published?
A: It is in the process. It is a very important study and one of the top journals in the field is currently reviewing it.

Q: Can you explain what peer review means?
A: No article can be published until other scientists in the field have read it and had a chance to ask questions, make you revise it, or to reject it.

Q: Why would an article not pass peer review?
A: If the methods used in the study are judged to be flawed by one's peers.

Q: But, there has been no opportunity for anyone to read your article and voice concerns about it?
A: No, that process has not been completed yet.

Q: Ok, let's go to another topic. Can you please remind us how your rats were exposed to the PCBs in your study?
A: Yes, the rats are given a large dose of PCBs and then observed and tested for cancer; the standard procedure in the field.

Q: The Plaintiff's witness testified that there is, in fact, a link between PCBs and cancer. However, he exposed his rats differently. He exposed his rats with smaller doses over a longer period of time….Why didn't you you do that?
A: Well, this is not standard in the field. This is how I have always exposed my rats.

Q: Do you feel this is more similar to the way Mark Stevens was exposed to PCBs?
A: No, he was exposed in small amounts over a longer period of time.

Q: So, had you exposed the rats the way Mark Stevens was exposed, you could have also found a link between PCBs and cancer?
A: I certainly have no idea what the results would be.

Q: No further questions, Your Honor.

**VERSION 4: High-quality plaintiff expert & low-quality defense expert with "*Peripheral only*" cross-examination:**

**Expert Witness #1 testimony for the Plaintiff: Dr. Thomas Fallon**
Question from Mark Stevens' (the plaintiff) lawyer: Doctor, would you please state your name for the record.
Expert witness for the plaintiff's answer: My name is Dr. Thomas Fallon.

Q: And what is your profession?
A: I am a Professor and Biochemist at the University of Indiana Medical School, specializing in the effects of carcinogens in animals.

Q: So, you do cancer research?
A: Yes.

Q: Doctor, can you tell us your educational background?
A: I graduated from Johns Hopkins in 1980 with a double major in biology and chemistry. I received my PhD in Biochemistry from University of Oregon in 1984. I am currently a biochemist at the University of Indiana Medical School. I head a laboratory in which I study the effects of PCBs and other carcinogens on animals. I teach graduate courses on the effects of chemicals on living organisms. I have published 42 articles on cancer in peer-reviewed journals, two books, and have made over 100 presentations at national conferences.

Q (To the judge): At this time, Your Honor, the Plaintiff, Mark Stevens, wishes to qualify Doctor Thomas Fallon as an expert in the field of biochemistry.

Judge: Does the Defendant, Keen Co., have any objections?

Defense Attorney: No, Your Honor.
Judge: You may proceed.

Q: So, Doctor Fallon, you do research on substances that cause cancer?
A: Yes. In my laboratory I study several carcinogens, including PCBs.

Q: So PCBs are carcinogenic.
A: Yes, PCBs cause cancer.

Q: In your opinion, did Mark Stevens contract cancer from his exposure to PCBs?
A: Yes, I believe he did.

Q: Can you explain to us the value of animal studies? Why do you use animals?
A: We use animals to study the effects of dangerous chemicals because we can't use people. Laboratory animals are also easy and inexpensive to grow and maintain, they grow up quickly and have short life-spans, therefore you are able to conduct life span experiments quickly.

Q: So how do you conduct this research?

Q: I see. Dr., can you tell the jury how you conduct your research and why you are so confident about this connection between PCBs and cancer?
A: I conduct experimental studies on animals to assess whether PCBs cause cancer.

Q: Could you please describe for us a specific study your lab has conducted and explain the results?
A: In one study, we randomly assigned a group of rats to receive a set dose of PCB, Arochlor 1254 was the exact type of PCB, while another group of rats did not receive the PCBs. We exposed the rats to PCBs for one month. Then we stopped and waited for a year. After a year, we compared the rates of cancer for the rats that ate PCBs and those in the control group that did not. We found that the rats exposed to PCBs had a significantly higher rate of liver cancer than those in the control group.

Q: Can you give the jury further details about how you go about conducting these experiments?
A: Of course. For the first phase of the study we exposed the rats to a small amount of PCBs every day for a prolonged period of time, similar to the exposure Mark Stevens would have undergone at Keen Company. The exposure was small, but steady, we exposed the rats every day for a month. The control group was exposed to a completely harmless dose of saline every day for a month. After this period was over, we observed the rats for a year, after which we tested for cancer and recorded the rate of occurrence.
Q: What exactly did you observe?
A: After one year, the rats who were exposed to PCBs had a significantly higher rate of liver cancer than the rats who were not exposed to PCBs. These results were published in *Neurology & Toxicology*.

Q: Doctor Fallon, how many studies like this have you conducted?
A: My lab has published about 42 papers. Out of those, we probably published 27 papers on animal studies, and probably 15 papers were on PCBs in particular.

Q: Doctor Fallon, everything you've told us here today suggests very strongly that colon cancer can result from exposure to PCBs. Is there anything else that you believe would support your statement?
A: Yes. I looked at others who worked at Keen Co. who developed cancer around the same time as Mark Stevens, and I found 47 people. Of those, seven workers were diagnosed with cancer. This is much more than would ever be expected by chance alone.

Q: Doctor, of these seven men at the Keen plant who got cancer, what kinds of cancer did they get?
A: There were two people with colon cancer, one person who had lung cancer, one person with liver cancer, and three other kinds.

Q: Doctor, why would one carcinogen cause so many kinds of tumors?
A: The men might have been exposed in different ways. That is, the PCBs might have come to these men through the air, the water, the soil, or through contact with their skin. So the PCBs might have gotten into the bodies in different ways, and that might have caused the different cancers.

Q: And there is no reason to believe that Mark would have contracted cancer had he not been exposed to PCBs?
A: That is also correct.

Q: Doctor Fallon, can you give us your expert opinion as to the cause of Mark's colon cancer?
A: In my expert opinion, Mark Stevens' colon cancer was caused by the exposure to PCBs at the Keen company plant.

Q: Thank you. That is all, Your Honor.

**Cross-examination of Dr. Fallon:**
Question from the defendant's (Keen Company) lawyer: Doctor Fallon, is it true that you are not a lawyer?
Dr. Fallon's answer: That is correct.

Q: So, you are not a legal expert who can testify as to whether the company is legally responsible for Mark Stevens' illness?
A: No, I cannot speak to that at all. I am not an expert about legal matters.

Q: So, your opinion as to whether Keen Co is responsible for Mark Steven's illness is irrelevant?
A: Well, my opinion about whether PCBs cause cancer is very relevant.

Q: Of course, but when you said during your testimony that it is your expert opinion that PCBs caused Mark Stevens' illness, that was somewhat misleading, wasn't it?
A: How so?

Q: Well, your research is about whether PCBs cause cancer generally, but your research was not specific to Mark Stevens' case, was it?
A: No, it was not.

Q: Can you predict any specific individual's likelihood of developing cancer as a result of PCBs from your study's results?
A: No, I cannot.

Q: So, how much were you paid to be here today?
A: I don't think that is relevant.

Q: You are, in fact, paid to be here today, is this correct?
A: Yes, I am. I am paid to educate people in many different ways, this is no exception. This is my job.

Q: And can you tell us who paid for you to be here today?
A: The plaintiff.

Q: And how often do you get paid to testify?

A: I do not do this for the money if that is what you are inferring. I do not need the money.

Q: Some experts testify for free to avoid that confusion, don't they?
A: Yes.

Q: But not you.
A: No. I do not testify for free, but many expert witnesses accept compensation for their testimony.

Q: How often do you testify, Dr. Fallon?
A: I typically testify a few times a year.

Q: How many times have you testified overall?
A: I have testified in 45 cases.

Q: Do you always testify for the plaintiff?
A: No, I do not…I have testified in cases as an expert for the plaintiff many times, but I have also testified for the defense in cases where I do not believe the defendant's illness was caused by PCBs.

Q: How many times has this happened – where you testify for the defense?
A: I have testified for the defense 7 times.

Q: So, you testify for the plaintiff side 84% of the time.
A: Yes, thus far.

Q: So, would you call yourself more of a plaintiff witness?
A: No, I would not. I testify for the side that I believe in.

Q: So, 84% of cases involving PCBs just happen to be strong plaintiff cases?
A: I believe it to be a dangerous substance. I am surprised there aren't more.

Q: No further questions, Your Honor.

**Expert Witness #2 for the Defense: Dr. William Campbell**
Judge: We are now ready to proceed with the Defense's witness. Are you ready with your witness?
Keen Co.'s (the defendant) lawyer: Yes, Your Honor.

Judge: You may proceed.

Question from Keen Co.'s lawyer: Doctor, would you please state your name and address for the record?
The expert witness for the defense's answer: My name is Dr. William Campbell.

Q: Doctor Campbell, what is your current occupation?

A: I am a professor of Biochemistry at the University of Michigan.

Q: Thank you. And were did you receive your training in this science?
A: I received my Bachelor's degree from Tufts University in 1977 with a concentration in mathematics and biology. I went on to complete my PhD studies in Biochemistry in 1982 at University of Iowa. I am currently a Professor of biochemistry at the University of Michigan. I teach a seminar in Biochemistry to graduate students in public health, and an "Environment and Disease" class to the medical students. I have published over 30 peer reviewed articles in such journals, a book and a number of invited chapters for edited books. I have also presented my work at national conferences on well over 100 occasions in the last 15 years.

Q (To the judge): At this time, Your Honor, the defendant, Keen Co. wishes to qualify Doctor William Campbell as an expert in the field of biochemistry.

Judge: Does the plaintiff, Mark Stevens have any objections?
Howard (plaintiff, Mark Stevens): No, Your Honor.
Judge: Mr. Moore, you may proceed.

Q: Doctor, you have conducted many studies on the effects of PCBs on populations. What have these studies indicated with respect to the association between PCBs and colon cancer?
A: I have found that exposure to PCBs does not cause any increase in the rates of colon cancer.

Q: So people who were exposed to PCBs were no more likely to contract colon cancer than anybody else in your studies?
A: That's right. My research has indicated that animals in contact with PCBs did not show a high incidence of cancer.

Q: But what does that mean for the plaintiff, Mark Stevens?
A: What that means is that it is unlikely his colon cancer came from PCBs.

Q: So in your expert opinion, PCBs did not cause Mark Stevens' colon cancer?
A: That's right. I believe that PCBs did *not* cause Mark Stevens' colon cancer.

Q: Is it correct to say that PCBs have not been shown to cause cancer, but they have not been ruled out as a cause of cancer?
A: I think it's stronger than that. There really is very little evidence that PCBs are related to the cancer problem. Although PCBs are poisonous and will kill a person immediately if digested in a large quantity, there is no evidence that PCBs cause cancer in humans.

Q: Could you please describe for us a specific study your lab has conducted and explain the results?
A: My studies expose animals to PCBs and observe the effects on the animals, more specifically whether or not they develop cancer. For example, one experiment involved a group of 15 rats who all received a large dose of Arochlor 1254 (the kind of PCB Mark Stevens was exposed to). We then observed the rats for a year to determine whether or not they developed cancer. We found a high cancer rate in rats exposed to PCBs.

Q: What were your results?
A: We did not find a high incidence of cancer in the rats. These results are currently under review for publication in *Neurology & Toxicology*.

Q: Are you aware that the plaintiff's witness is testifying that his study found contrary results? How do you explain the fact that his animal study found that PCBs caused cancer while your study did not?
A: Animal studies are only useful when one is only talking about the same kind of cancer in humans. For example, it would be reasonable to use a study's findings that PCBs caused liver cancer in mice to say that humans may also be afflicted with liver cancer. But we can only do this when we are talking about the same type of cancer. You can't compare across different cancers.

Q: So, if a study found that PCBs caused liver cancer in rats, it would be improper in your opinion, to say that PCBs caused colon cancer in humans?
A: Exactly. Just because PCBs might cause liver cancer in rats, you can't say whether or not PCBs might cause colon cancer in humans. They're two different organs and species, it's just not the same.

Q: Then, in your opinion, would you say that PCBs were not the cause of the plaintiff, Mark Stevens' illness?
A: Yes, it is very unlikely that PCBs caused the plaintiff, Mark Stevens' colon cancer.

Q: Thank you very much, Doctor.

**Cross-examination of Dr. Campbell:**
Judge: You may cross-examine the witness.

Mark Stevens' lawyer: Thank you, Your Honor.

Question from Mark Stevens' lawyer: Dr. Campbell, do you always testify for the defense?
Dr. Campbell's answer: No, I do not… I have testified for the plaintiff side before.

Q: Why would you do that, if your studies show that PCBs do not cause cancer?
A: Well, occasionally there are cases where it is obvious that the person's illness was caused by PCBs.

Q: Would you say it is important for an expert to not always side with the same side in a court case?
A: Yes, I would agree with that.

Q: Why do you feel that is important?
A: Well, one does not want to become a hired gun for one side…an expert should stay balanced.

Q: And do you consider yourself balanced?

A: Well, I'd like to find more cases for the plaintiff to testify for, but I find that I tend to mostly find cases in which I believe in the defense's side.

Q: How many times have you testified for the plaintiff?
A: 10 times.

Q: Out of how many?
A: Roughly 90 cases.

Q: So, you have testified for the defense 90% of the time?
A: That is correct. There aren't many cases in which I believe PCBs caused the cancer.

Q: Doctor Campbell, isn't it true that your research has been criticized by your peers?
A: No, that is not true. I am very well respected in my field.

Q: Are you saying that there is no dispute in your field about the accuracy of your study that found that PCBs cause cancer?
A: Well, some scientists do disagree with me, but that is not based on the quality of my work.

Q: What would it be based on then?
A: Different researchers conduct their research differently and as such, they find different results. All researchers find themselves needing to defend their methods and their conclusions at some point or another. I have not had to do so any more than any other researcher in the field.

Q: How can you be sure that your way is correct and that those who find different results are wrong?
A: I stand by my methods and my research… as do the many journals and publishers that have published my work. Articles must go through a rigorous peer review process before they are published. If my methods were flawed, I would not have been published.

Q: Would you say that everything that is published should be trusted?
A: No, there are some lower level journals that have lower standards.

Q: Are all of your studies published in the top journal in the field?
A: No, of course not all of them, but some of them are. I can assure you, all the journals I publish in are respected journals that can be trusted.

Q: Are you paid to testify today doctor?
A: Yes, of course. All experts are offered compensation for their testimony. We are providing a valuable service to the court.

Q:And is it the court that pays you?
A: No, it is not.

Q: Who pays you then?
A: The defense pays me.

Q: No further questions, Your Honor.

**Measures**

Need for Cognition Scale

Below is a list of statements that some people use to describe themselves. Read each statement and decide how much you think it describes you. There are no right or wrong answers. Use the following scale:

| 1 | 2 | 3 | 4 | 5 |
|---|---|---|---|---|
| **Extremely Uncharacteristic** | **Somewhat Uncharacteristic** | **Uncertain** | **Somewhat Characteristic** | **Extremely Characteristic** |

| | | | | | |
|---|---|---|---|---|---|
| 1 | 2 | 3 | 4 | 5 | 1. I would prefer complex to simple problems. |
| 1 | 2 | 3 | 4 | 5 | 2. I like to have the responsibility of handling a situation that requires a lot of thinking. |
| 1 | 2 | 3 | 4 | 5 | 3. Thinking is not my idea of fun. |
| 1 | 2 | 3 | 4 | 5 | 4. I would rather do something that requires little thought than something that is sure to challenge my thinking abilities. |
| 1 | 2 | 3 | 4 | 5 | 5. I try to anticipate and avoid situations where there is likely chance that I will have to think in depth about something. |
| 1 | 2 | 3 | 4 | 5 | 6. I find satisfaction in deliberating hard and for long hours. |
| 1 | 2 | 3 | 4 | 5 | 7. I only think as hard as I have to. |
| 1 | 2 | 3 | 4 | 5 | 8. I prefer to think about small, daily projects to long-term ones. |
| 1 | 2 | 3 | 4 | 5 | 9. I like tasks that require little thought once I've learned them. |
| 1 | 2 | 3 | 4 | 5 | 10. The idea of relying on thought to make my way to the top appeals to me. |
| 1 | 2 | 3 | 4 | 5 | 11. I really enjoy a task that involves coming up with new solutions to problems. |
| 1 | 2 | 3 | 4 | 5 | 12. Learning new ways to think doesn't excite me very much. |
| 1 | 2 | 3 | 4 | 5 | 13. I prefer my life to be filled with puzzles that I must solve. |
| 1 | 2 | 3 | 4 | 5 | 14. The notion of thinking abstractly is appealing to me. |
| 1 | 2 | 3 | 4 | 5 | 15. I would prefer a task that is intellectual, difficult, and important to one that is somewhat important but does not require much thought. |
| 1 | 2 | 3 | 4 | 5 | 16. I feel relief rather than satisfaction after completing a task that requires a lot of mental effort. |
| 1 | 2 | 3 | 4 | 5 | 17. It's enough for me that something gets the job done; I don't care how or why it works. |
| 1 | 2 | 3 | 4 | 5 | 18. I usually end up deliberating about issues even when they do not affect me personally. |

*Note*: items 3, 4, 7, 8, 9, 12, 16, and 17 were reverse-scored.

Is the defendant, Keen Company "not negligent" (i.e., Keen Company **is not** responsible for Mark Stevens' cancer) or "negligent" (i.e., Keen Company **is** responsible for Mark Stevens' cancer)? Please circle **ONE**:

NOT NEGLIGENT NEGLIGENT

How confident are you in your verdict? Please circle **ONE**:

| 0% | 10% | 20% | 30% | 40% | 50% | 60% | 70% | 80% | 90% | 100% |
|---|---|---|---|---|---|---|---|---|---|---|
| Not at all Confident | | | | | | | | | | Completely Confident |

**Dr. Fallon** testified as an expert witness for **the plaintiff** (Mark Stevens). **Based on the testimony and evidence,** how credible was Dr. Fallon? Please circle **ONE**:

| -3 | -2 | -1 | 1 | 2 | 3 |
|---|---|---|---|---|---|
| Very **Not** Credible | **Not** Credible | Somewhat **Not** Credible | Somewhat Credible | Credible | Very Credible |

**Dr. Campbell** testified as an expert witness for **the defense** (Keen Company). **Based on the testimony and evidence,** how credible was Dr. Campbell? Please circle **ONE**:

| -3 | -2 | -1 | 1 | 2 | 3 |
|---|---|---|---|---|---|
| Very **Not** Credible | **Not** Credible | Somewhat **Not** Credible | Somewhat Credible | Credible | Very Credible |

**GROUP VERDICT FORM**

Your jury's group verdict choices are: (Please circle **ONE** verdict when your jury has reached a **UNANIMOUS** decision):

We, the jury:

FIND THE DEFENDANT **NOT NEGLIGENT**

FIND THE DEFENDANT **NEGLIGENT**

| **How <u>persuasive</u> was this juror during deliberations?** | **Not at all Persuasive** | | | | | **Very Persuasive** |
|---|---|---|---|---|---|---|
| You (Juror # 4) | 1 | 2 | 3 | 4 | 5 | 6 |
| Juror # 1 | 1 | 2 | 3 | 4 | 5 | 6 |
| Juror # 2 | 1 | 2 | 3 | 4 | 5 | 6 |
| Juror # 3 | 1 | 2 | 3 | 4 | 5 | 6 |
| Juror # 5 | 1 | 2 | 3 | 4 | 5 | 6 |
| Juror # 6 | 1 | 2 | 3 | 4 | 5 | 6 |

Please choose the race that you identify with:

_____American Indian or Alaska Native

_____Hispanic or Latino

_____Asian

_____Black or African American

_____Native Hawaiian or Other Pacific Islander

_____White

_____Other

Age: _________________________________

Gender:______________________________
